# Machine Learning-Assisted Drug Repurposing Framework for Discovery of Aurora Kinase B Inhibitors

**DOI:** 10.3390/ph18010013

**Published:** 2024-12-25

**Authors:** George Nicolae Daniel Ion, George Mihai Nitulescu, Dragos Paul Mihai

**Affiliations:** Faculty of Pharmacy, “Carol Davila” University of Medicine and Pharmacy, Traian Vuia 6, 020956 Bucharest, Romania; george-nicolae-daniel.ion@umfcd.ro (G.N.D.I.); dragos_mihai@umfcd.ro (D.P.M.)

**Keywords:** AURKB, cancer therapy, protein kinase inhibition, virtual screening, computer-aided drug design and discovery, interaction fingerprints, QSAR, classification models

## Abstract

**Background:** Aurora kinase B (AurB) is a pivotal regulator of mitosis, making it a compelling target for cancer therapy. Despite significant advances in protein kinase inhibitor development, there are currently no AurB inhibitors readily available for therapeutic use. **Methods:** This study introduces a machine learning-assisted drug repurposing framework integrating quantitative structure-activity relationship (QSAR) modeling, molecular fingerprints-based classification, molecular docking, and molecular dynamics (MD) simulations. Using this pipeline, we analyzed 4680 investigational and approved drugs from DrugBank database. **Results:** The machine learning models trained for drug repurposing showed satisfying performance and yielded the identification of saredutant, montelukast, and canertinib as potential AurB inhibitors. The candidates demonstrated strong binding energies, key molecular interactions with critical residues (e.g., Phe88, Glu161), and stable MD trajectories, particularly saredutant, a neurokinin-2 (NK2) antagonist. **Conclusions:** Beyond identifying potential AurB inhibitors, this study highlights an integrated methodology that can be applied to other challenging drug targets.

## 1. Introduction

Cancer remains one of the leading causes of mortality worldwide, with millions of new cases and deaths reported annually [1]. Despite significant advances in cancer therapy, drug resistance continues to pose a substantial challenge, driving the ongoing search for new treatments [2]. This underscores the importance of identifying novel therapeutic targets and developing new drugs to enhance the efficacy of cancer treatments and overcome resistance.

Protein kinases have emerged as pivotal targets in cancer therapy due to their crucial roles in regulating cellular processes such as proliferation, differentiation, and apoptosis. These enzymes function by transferring phosphate groups to specific substrates, an essential process for signaling pathways that control cell growth and survival. Aberrations in kinase activity, whether through overexpression, mutation, or dysregulation, are commonly associated with cancer, making these enzymes attractive targets for cancer drug design [3].

Among the plethora of protein kinases implicated in cancer, Aurora kinases have drawn considerable attention. Aurora kinases are a family of serine/threonine kinases comprising isoforms A, B, and C. These kinases are essential regulators of mitosis, ensuring accurate chromosomal segregation and cytokinesis [4,5]. Aurora kinase B (AurB) is particularly critical as it plays a central role in the chromosomal passenger complex (CPC), which is vital for correcting kinetochore-microtubule attachments, regulating the mitotic spindle checkpoint, and ensuring proper chromosomal alignment and segregation during cell division [5,6]. The CPC, which includes the proteins AurB, INCENP (inner centromere protein), survivin, and borealin, orchestrates these processes, highlighting the indispensable function of AurB in maintaining genomic stability [5,7].

AurB overexpression and hyperactivation have been linked to various malignancies, including colorectal, breast, prostate, ovarian, testicular, thyroid, and lung cancers, often correlating with poor prognosis and resistance to therapy [6,8]. This overexpression disrupts normal mitotic processes, causing defects such as multipolar spindles, chromosomal mis-segregation, and cytokinesis failure, which drive tumor progression and aneuploidy—a hallmark of cancer [8,9,10]. Moreover, AurB contributes to tumor cell survival by enhancing DNA damage tolerance and activation of pro-survival pathways [4,8,11]. As a result, AurB inhibitors have gained attention for their potential to selectively induce mitotic catastrophe and apoptosis in cancer cells [8,12,13,14].

The development and testing of several AurB inhibitors have been described in the literature, and they have had varying degrees of success. Barasertib (AZD1152), an ATP-competitive inhibitor, has shown high selectivity for AurB and efficacy in preclinical models, inducing mitotic defects and apoptosis in acute myeloid leukemia (AML) and lung cancer [15,16]. Other ATP-competitive inhibitors, such as hesperadin and ZM447439, also disrupt mitosis and cytokinesis, leading to polyploidy and cancer cell death [16,17]. Allosteric inhibitors, targeting non-ATP-binding sites, and dual inhibitors like VX-680 and GSK1070916 offer alternative strategies, though challenges remain in developing these compounds due to structural complexities and specificity requirements [16,18,19].

Despite these advancements, developing selective and potent AurB inhibitors remains challenging. The structural similarity between AurB and its closely related isoform Aurora A (AurA) complicates the design of highly specific inhibitors, as they share a conserved catalytic domain. Achieving high selectivity is crucial to minimize off-target effects such as cytotoxicity and hematological toxicity [8,20,21]. Drug resistance is another important obstacle in AurB-targeted therapy, as cancer cells can adapt through various mechanisms, including activation of compensatory pathways or mutations that alter the binding affinity of inhibitors [4,16,22]. For instance, mutations in the AURKB gene, such as Gly160Glu, can reduce inhibitor binding efficiency, diminishing therapeutic efficacy [22,23].

Advances in structure-based drug design, including high-throughput screening and virtual docking, have been instrumental in identifying new AurB inhibitors. However, optimizing these compounds for high specificity and overcoming resistance mechanisms remains a critical challenge. In silico drug discovery methods, such as molecular docking and quantitative structure-activity relationship (QSAR) analysis, can accelerate the identification of lead compounds, but further refinement is needed to translate these findings into clinical therapies [16,24].

Structurally, AurB comprises three main domains: an N-terminal regulatory domain, a highly conserved kinase domain, and a short C-terminal extension. The kinase domain consists of a β-stranded lobe and an α-helical lobe connected by a hinge region, critical for its catalytic AurB. The phosphorylation of the activation loop, particularly at Thr232, is essential for AurB activation [8,24,25].

As proven in other studies, key residues within the ATP-binding pocket include Leu83, Phe88, Glu155, and Ala157, which contribute significantly to substrate recognition and inhibitor binding. The hinge region and the conserved T-loop are also important for modulating kinase activity. The structural flexibility of these regions allows AurB to interact with various inhibitors, though this also complicates the design of selective inhibitors due to the overlap with AurA binding sites [20,26].

In order to improve affinity while also increasing selectivity for AurB isoform, the interaction of known ligands and inhibitors with the target protein can be studied form the perspective of chemical structure and amino acid interactions, as detailed information may aid in the development or selection of more potent and selective drug candidates.

Molecular interaction fingerprints (MIFs) are computational representations of protein–ligand interactions that capture critical contact points within a binding site. These fingerprints map interactions such as hydrogen bonds, hydrophobic contacts, π–π stacking, and electrostatic forces, offering a detailed profile of binding affinity. Utilizing MIFs can significantly enhance post-docking analysis by allowing researchers to quantitatively assess the strength and nature of interactions between the targeted protein and potential inhibitors. This technique goes beyond traditional scoring functions by identifying key residues involved in binding and predicting the impact of structural modifications on inhibitor efficacy [26,27,28,29].

By integrating analysis of interaction profiles predicted with molecular docking and machine learning-based approaches (ML), the current study aims to develop a drug repurposing framework to facilitate the identification of structural features that contribute to high binding affinity towards AurB and to discover novel potential inhibitors among approved or investigational drugs.

## 2. Results

### 2.1. Datasets

The initial dataset contained 179 inhibitors tested on the AurB/INCENP complex, as retrieved from the ChEMBL database. Of these, 127 compounds presented exact half maximal inhibitory concentration values (IC50) available and were retained for further analysis. The pIC50 values representing the negative logarithm (base 10) of the IC50 were further used for subsequent analysis. This subset, referred to as the “exact values set” (EV set), served as the basis for scaffold structure analysis and QSAR modeling. Supplementary Material Table S1 illustrates descriptive statistics for the total and EV sets of AurB inhibitors, including several molecular descriptors calculated with DataWarrior v06.03.01 software [29] based on their chemical structure.

The EV set exhibited diverse physicochemical properties, with molecular weights ranging from approximately 198.2 to 663.0 g/mol and pIC50 values spanning from 4.0 to 9.3.

The 550 decoy compounds generated with the DUD-E platform presented a comparable profile of molecular descriptors to the inhibitors, supporting their suitability as decoy molecules. This dataset exhibits a similar range of molecular weights, AlogP values, number of H-bond acceptors and H-bond donors, and other descriptors, as detailed in Supplementary Material Table S2.

### 2.2. Scaffold Analysis

Using the EV set, scaffold analysis was performed to identify the most central ring (MCR) systems associated with the compounds. The 127 compounds yielded 11 distinct MCR fragments, numbered from 1 to 11. Scaffolds 10 and 11, each represented by a single compound, were excluded from further analysis due to limited statistical significance. Additionally, MCR1 and MCR2, each comprising two bioisosteric compounds, the pyrazole and furane rings, were grouped into a single category (MCR1 + 2), resulting in a final set of eight scaffold categories for further evaluation.

**Figure 1:**
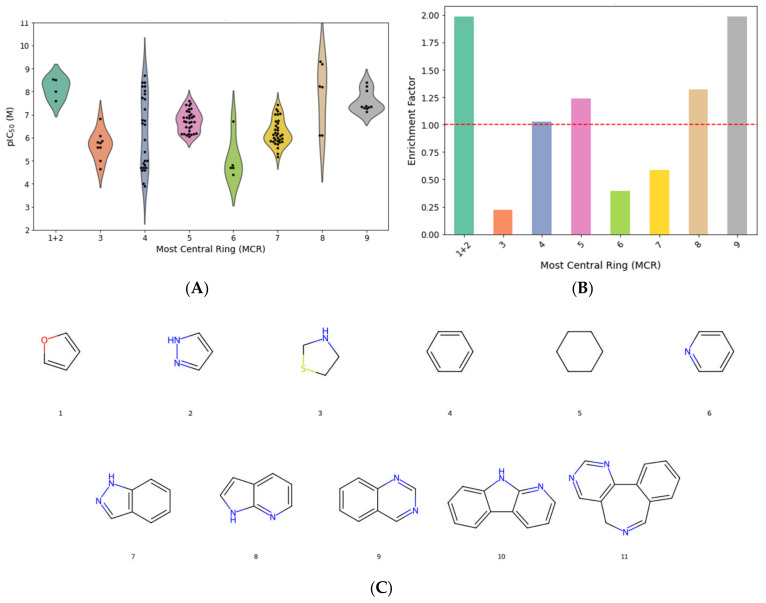
Scaffold analysis of AurB inhibitors with exact values for IC50 (n = 127). (**A**) Violin plots illustrating pIC50 values distribution among most central ring systems (MCR); (**B**) enrichment factors by MCR with the dotted red line representing a frequencies ratio of 1 (no enrichment); (**C**) structures of all MCR scaffolds generated based on studied AurB inhibitors structures (MCR10 and MCR11 excluded from statistical analysis, as explained in Section 4.2).

In order to evaluate the relationship between these scaffolds and inhibitor potency (pIC50), Mann–Whitney U tests and Chi-squared tests were performed (results shown in Supplementary Material, Table S3). The pIC50 threshold of 6.5 was selected to divide the compounds into two subsets, distinguishing potent inhibitors from weaker ones. Significant results were observed for MCR1 + 2, MCR8 (pyrrolo [2,3-b]pyridine), and MCR9 (quinazoline), which showed both high enrichment factors (EF) and statistically significant associations with increased potency (Figure 1A–C). MCR1 + 2 exhibited a mean pIC50 value of 8.15 (*p* = 0.0038), underscoring its potential as a scaffold associated with enhanced activity.

The Chi-squared test yielded a value of 29.7068 (*p* = 0.0001), indicating statistically significant differences in the distribution of high-potency inhibitors across the scaffolds. Significant residuals were observed for MCR9, suggesting this scaffold was disproportionately represented among the most potent inhibitors.

The EF analysis further highlighted trends in scaffold potency. MCR1 + 2, MCR5, MCR8, and MCR9 displayed EF values greater than 1, indicating a higher-than-expected proportion of potent inhibitors within these scaffolds. However, MCR5 (cyclohexane), despite showing an EF > 1, did not exhibit statistically significant differences in mean pIC50 values when compared to the other scaffolds, suggesting that while MCR5 contains potent inhibitors, its contribution to potency may not be as robust or consistent as that of MCR1 + 2, MCR8, and MCR9.

### 2.3. QSAR Models

Several quantitative structure–activity relationship (QSAR) models were developed using the exact values set to predict pIC50 values based on selected molecular descriptors generated with RDKit (as described in the Section 4). The molecular descriptors selected using the implemented feature selection approaches are explained in Table 1, while the heatmap of intercorrelations is shown in Supplementary Materials Figure S1. The highest negative correlation was observed between SPS and BCUT2D_CHGLO (r = −0.83), while the highest positive correlation was noted between SPS and VSA_EState3 (r = 0.74). Most of the absolute values of correlation coefficients are below 0.7, highlighting a relatively low degree of collinearity between the selected features.

Five regression models—Multiple Linear Regression (MLR), Partial Least Squares (PLS), Support Vector Machines (SVM), Random Forest (RF), and Gradient Boosting (GB)—were trained and evaluated after splitting the dataset into training (80%) and test (20%) subsets. The performance metrics for the trained models, including R^2^, RMSE, MAE, and Q^2^ cross-validation values, are summarized in Table 2.

Supplementary Material Figure S2 shows the correlation diagrams between experimental and the pIC50 values predicted by all five models, further illustrating the poor predictive performance of linear models (MLR and PLS) compared to non-linear methods (SVM, RF, and GB) in accordance with the corresponding correlation coefficients.

The SVM model demonstrated the best overall performance, achieving a cross-validated Q^2^ of 0.67 ± 0.10 and a test set R^2^ of 0.81. The RF and GB models also demonstrated strong test R^2^ values (~0.76) but showed evidence of higher overfitting, as reflected by their exceptionally high training R^2^ values (0.9587 for RF and 0.9949 for GB).

**Figure 2:**
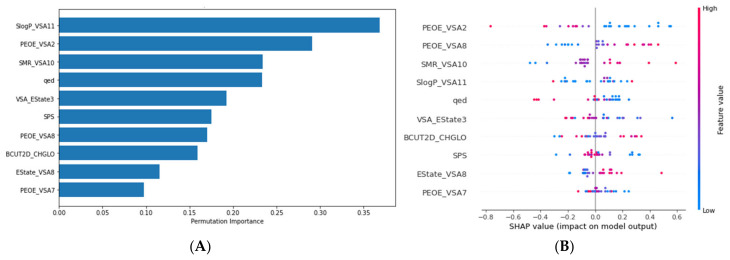
Feature importance analysis for the selected SVM model. (**A**) Permutation importance; (**B**) Shapley Additive explanations (SHAP) analysis plot showing feature impact on model output (test set compounds).

Feature importance computed by permutation approach for the SVM model revealed that molecular descriptors such as SlogP_VSA11, PEOE_VSA2, SMR_VSA10, and qed had the most significant contributions to model predictions (Figure 2A). The SHAP (Shapley additive explanations) summary plot (Figure 2B) shows that, on average, lower values for PEOE_VSA2, qed, VSA_Estate3, and PEOE_VSA7 are required for higher predicted potencies. On the other hand, high values for PEOE_VSA8, SMR_VSA10, and EState_VSA8 are correlated with higher AurB inhibitory activity. However, the dependency between SlogP_VSA11, BCUT2D_CHGLO, SPS, and predicted pIC50 is less linear, highlighting the capacity of the SVM model to capture non-linear relationships. Partial dependence and individual conditional expectation plots are shown in Supplementary Material Figure S3A–J, further illustrating the influence of each descriptor on the predicted pIC50 value.

According to the SHAP analysis, compounds with a lower van der Waals surface area (VSA) of atoms with more electronegative partial charges (PEOE_VSA2) and with partial charges close to 0 (PEOE_VSA7) are more likely to be potent inhibitors, while molecules with lower VSA of atoms with partial charges between 0 and 0.05 (PEOE_VSA8) are less active. PEOE is a method of partial equalization of orbital electronegativities for calculating atomic partial charges, accounting for the differences in electronegativity among bonded atoms. Furthermore, compounds with atoms with high contributions to molar refractivity (SMR_VSA10) are also correlated with higher activities, as well as atoms with high EState indices (EState_VSA8). Additionally, molecules with lower sums of EState indices for atoms with relatively low VSA contributions (VSA_EState3) are also more potent. Interestingly, lower drug-likeness scores (qed) are associated with higher activities, while higher spatial complexity scores (SPS) are specific to less active compounds. Additional insights into the QSAR models are provided in the Supplementary Material. Figure S4 illustrates the applicability domain of the SVM model through a Williams plot and principal component analysis (PCA). Most test set compounds fall within the reliable prediction domain, validating the model’s robustness. Table S4 outlines the range of values for selected molecular descriptors in the training and test sets, showing sufficient overlap to ensure that the model was tested on a representative chemical space.

### 2.4. Fingerprints Classification Models

**Figure 3:**
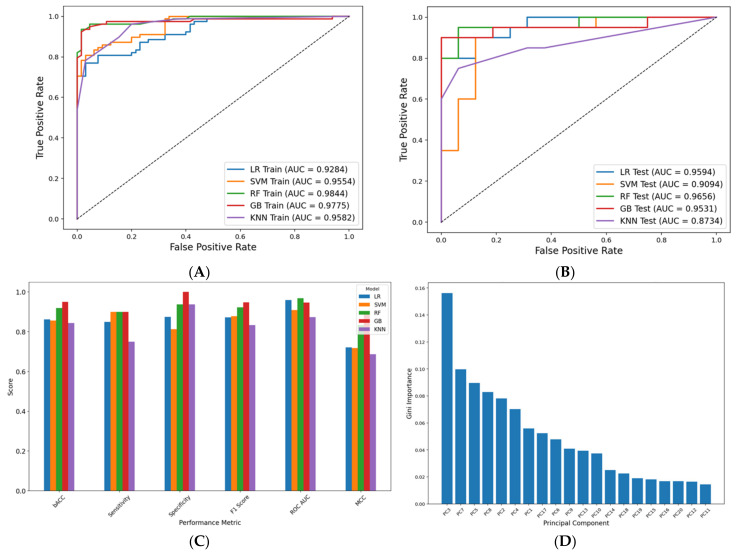
Analysis of performance for classification models based on flexophore fingerprints. (**A**) ROC curves for the training set; (**B**) ROC curves for the test set; (**C**) performance metrics for the test set; (**D**) feature importance scores (Gini importance) for the trained RF model. LR—logistic regression; SVM—support vector machine; RF—random forest; GB—XGBoost; KNN—K-nearest neighbors.

Classification models were developed using 20 principal components (PCs) of flexophore fingerprints to distinguish active (pIC50 > 7) from inactive inhibitors. Performance metrics for logistic regression (LR), support vector machine (SVM), random forest (RF), gradient boosting (GB), and K-nearest neighbors (KNN) classifiers are presented in Figure 3C, with additional data in Supplementary Materials Table S5.

RF and GB models outperformed the other classifiers, with training accuracies of 97.2% and 94.4%, respectively, and test accuracies exceeding 90%. ROC curves for the test set confirmed robust model performance, with test set ROC AUC values of 0.9688 for RF and 0.9469 for GB. The RF classifier was selected for downstream analysis based on its ability to accurately identify active ligands while minimizing false positives and its higher values for cross-validation performance metrics. The Gini importance scores of the 20 selected PCs for the RF model are shown in Figure 3D. Notably, PC3 has a significantly larger contribution to predictions compared to the other PCs.

### 2.5. Ligand-Based Meta-Model

**Figure 4:**
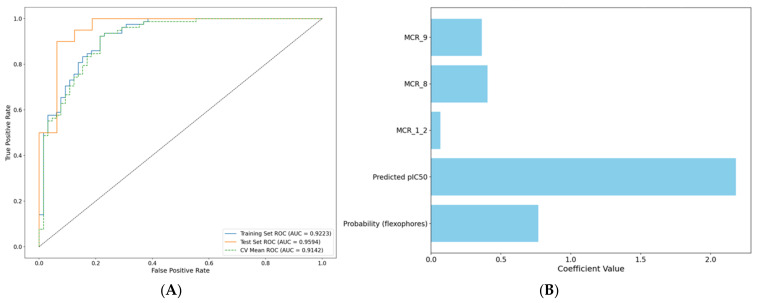
Analysis of the trained logistic regression meta-model. (**A**) ROC curves for test, training sets, and five-fold cross-validations; (**B**) regression coefficients for the used variables.

A stratified logistic regression meta-model was developed to integrate scaffold-specific information (one-hot encoded MCR scaffolds), predicted pIC50 values (from the selected QSAR model), and predicted probabilities (from the flexophore-based classification model) for distinguishing active from inactive AurB inhibitors (pIC50 > 7). Figure 4 illustrates the performance of the meta-model, presenting the ROC curves for the training, test, and cross-validation datasets (Figure 4A), as well as the regression coefficients for the features used in the model (Figure 4B).

The meta-model demonstrated strong predictive performance, achieving a ROC AUC of 0.9922 on the training set, 0.9594 on the test set, and a mean RIC AUC of 0.9142 across five-fold cross-validations. These results indicate strong discriminatory power and generalizability of the meta-model. Among the input features, pIC50 values predicted by the QSAR modeling and flexophore-based classification probabilities contributed the most, as evidenced by their higher regression coefficients (Figure 4B). This suggests that the integration of QSAR predictions and molecular fingerprints effectively captures key aspects of ligand activity. MCR1 + 2 and MCR9 also exhibited positive regression coefficients, reinforcing the relevance of these scaffolds in defining active AurB inhibitors.

### 2.6. Docking-Based Classification

**Figure 5:**
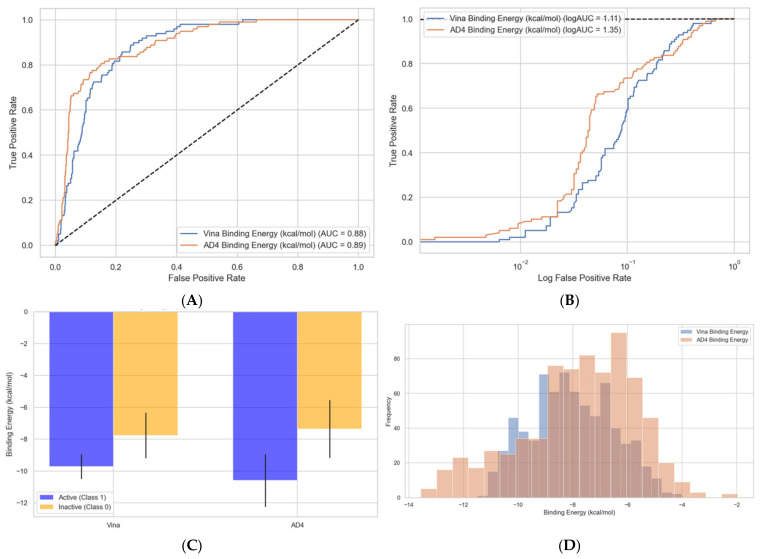
Comparison between binding energies predicted with AutoDock Vina 1.1.2 and AutoDock 4. (**A**) ROC curves for separating inactives from actives by binding energy values; (**B**) enriched ROC curves showing better separation for AutoDock 4 algorithm; (**C**) bar chart showing mean ± SD values for predicted binding energies by group and docking method; (**D**) histogram illustrating the distribution of predicted binding energies by method.

Docking studies using AutoDock Vina 1.1.2 and AutoDock 4 (AD4) were conducted on AurB inhibitors and decoy molecules. Redocking the co-crystallized structure of AurB inhibitor VX-680 showed that AD4 is more accurate than Vina in correctly predicting the binding pose in this specific case (RMSD of 1.8497 vs. 2.3386 Å, Supplementary Materials Figure S5). AD4 outperformed Vina in terms of accuracy and reliability, achieving higher ROC AUC values for distinguishing active inhibitors from inactive/decoys (Figure 5A,B). Additionally, AD4 demonstrated a better correlation between predicted binding energies and experimental pIC50 values, as illustrated in Supplementary Material Figure S6A,B.

Binding energy distributions and enriched ROC curves highlighted the ability of AD4 to prioritize potent inhibitors, with statistically significant differences (*t*-Student’s test, *p* < 0.0001) in binding energies between active and inactive groups (Figure 5C,D). Moreover, the effectiveness of the docking protocol was also evaluated by computing the enrichment factors in the top 1% and 10% of the dataset. For the top 1%, we determined enrichment factors of 2.13 for Vina and 3.19 for AD4, while at 10%, the values were 3.72 for Vina and 4.75 for AD4, the latter showing higher performance in correctly identifying active molecules.

**Figure 6:**
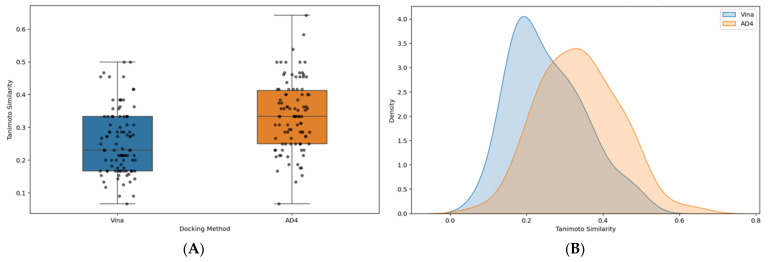
Comparison of calculated Tanimoto similarity indices between the docking algorithms. (**A**) Box plot illustrates mean and CI values for Tanimoto similarity indices; (**B**) KDE plot shows the distribution of Tanimoto similarity indices by docking method.

To further assess the performance of the docking algorithms, Tanimoto similarity indices were calculated and compared for the binding poses generated by Vina and AD4 (Figure 6). More specifically, Tanimoto similarity indices were calculated to quantitatively compare the molecular interaction fingerprints of docked ligands against the reference co-crystallized ligand (VX-680), reflecting the degree of overlap between predicted and reference interaction profiles, ranging from 0 (no similarity) to 1 (perfect match). By assessing this metric, we evaluated the fidelity of docking algorithms in reproducing experimentally validated binding interactions. The docking software that achieves higher Tanimoto similarity indices demonstrates a closer match to the reference interaction profile, indicating superior predictive accuracy. Figure 6A illustrates that, in our study, AD4 achieves a higher median Tanimoto similarity score compared to Vina, indicating improved consistency in docking pose accuracy. In contrast, Vina demonstrated less robust pose predictions. Figure 6B further supports these findings, where AD4’s distribution is shifted toward higher similarity indices, signifying a stronger overlap between predicted and reference poses. These results confirm AD4 as the superior docking algorithm in reproducing interaction patterns relevant for active AurB inhibitors, demonstrating the reliability of AD4 in generating biologically relevant binding poses through a quantitative and reproducible measure. Therefore, the selection of interaction fingerprints for downstream machine-learning models was performed based on the AD4 predictions.

**Figure 7:**
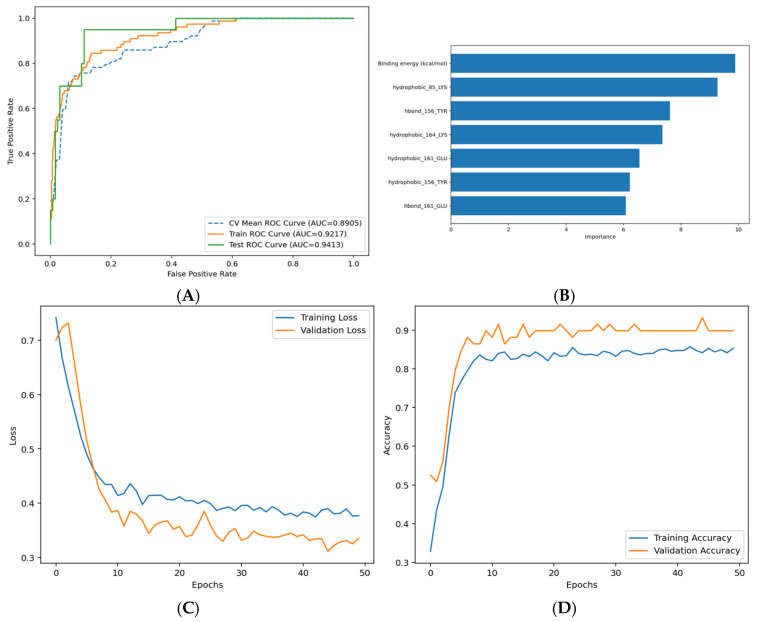
Analysis of trained MLP model. (**A**) Loss curves representing the error over time for training and test (validation) sets; (**B**) accuracy curves showing the proportion of correct predictions for training and test (validation) sets; (**C**) ROC curves for training, test sets, and 10-fold cross-validation; (**D**) importance values for the selected features.

A random forest classifier and Chi-square test were performed to select the most relevant interactions as features for training the docking-based ML model. Based on the computed importance scores, we selected the presence of hydrophobic interactions with Lys85, Tyr156, Glu161, and Lys164 and hydrogen bonds with Tyr156 and Glu161 as common features identified using both approaches. The feature importance scores for the top 10 interactions detected by both methods and the proportions of the common interactions in both active and inactive compounds are shown in Figure S7. Binding energies and the selected interaction fingerprints obtained using AD4 were thereafter used to train and fine-tune multiple multi-layer perceptron (MLP) classification models, leading to a total of 64 hyperparameter combinations. The most optimal MLP model was trained with a learning rate of 0.001, a batch size of 8.0, and a dropout rate of 0.1. The training, test, and cross-validation ROC curves, feature weights, loss curves, and accuracy curves for the selected MLP model are shown in Figure 7. The model achieved robust performance across multiple evaluation metrics. The training and test loss curves (Figure 7C) demonstrate consistent convergence, with no significant overfitting observed. Accuracy curves (Figure 7D) highlight the model’s ability to maintain high prediction accuracy for both training and test sets. Additionally, ROC curves (Figure 7A) indicate strong discrimination between active and inactive inhibitors, with test AUC values exceeding 0.95. The importance of specific interaction features in driving inhibitory activity predictions is visualized in Figure 7B.

As expected, binding energy stands out as the primary determinant, emphasizing its high contribution to ligand activity. Hydrophobic interactions with residues such as Lys85, Tyr156, Lys164, and Glu161 are highly ranked, highlighting the importance of hydrophobic contacts in stabilizing ligand binding within the active site. Hydrogen interactions involving Tyr156 and Glu161 residues are also noteworthy, as they are important for forming key hydrogen bonds and polar contacts.

### 2.7. Drug Repurposing Solutions

After predicting AurB inhibitory activity using molecular docking results as independent variables in the MLP model, we further analyzed compounds from DrugBank (investigational and approved drug sets encompassing 4680 molecules). Following applicability domain verification and similarity filtering using flexophore descriptors, 1024 compounds were selected. After further filtering through QSAR-based classification and meta-model analysis, 548 candidates were predicted to possess inhibitory activity against AurB and were further chosen for docking simulations.

Among the compounds with potential AurB inhibitory activity, 30 candidates with probabilities exceeding 95% were ranked and further analyzed (Table S6 in Supplementary Material). Notably, saredutant, montelukast, and canertinib emerged as the most promising repurposing candidates based on docking results and predicted probabilities.

Saredutant, an investigational neurokinin-2 (NK2) antagonist, demonstrated a predicted binding energy of −11.40 kcal/mol, a 57% probability of being active based on flexophore fingerprints, and a predicted pIC50 of 6.80 M. While its structure lacked a relevant MCR scaffold, the ligand-based meta-model predicted an 85.72% probability of activity, suggesting its potential for repurposing.

Montelukast, a leukotriene receptor antagonist used in asthma management, also lacked any relevant MCR scaffold. It showed a predicted binding energy of −11.10 kcal/mol, a flexophore-based predicted probability of activity of 78%; a predicted pIC50 of 6.35 M, and a meta-model predicted probability of 75.77%, also being suitable as a potential repurposing candidate.

Canertinib, an investigational EGFR, HER2, and ErbB-4 inhibitor, featured a quinazoline core scaffold (MCR9) and had a predicted binding energy of −10.39 kcal/mol. Despite a lower flexophore-based probability of activity (41%), it exhibited a high predicted pIC50 of 8.10 M and an exceptional meta-model probability of 99.63%, underscoring its potential as an AurB kinase inhibitor.

**Figure 8:**
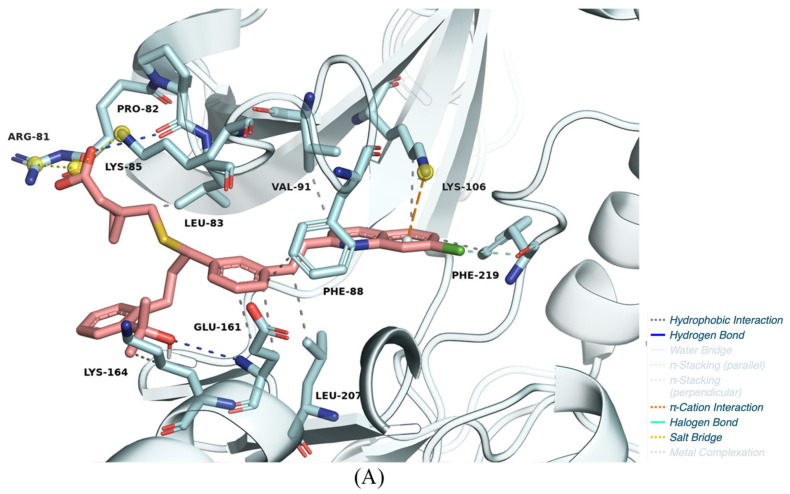

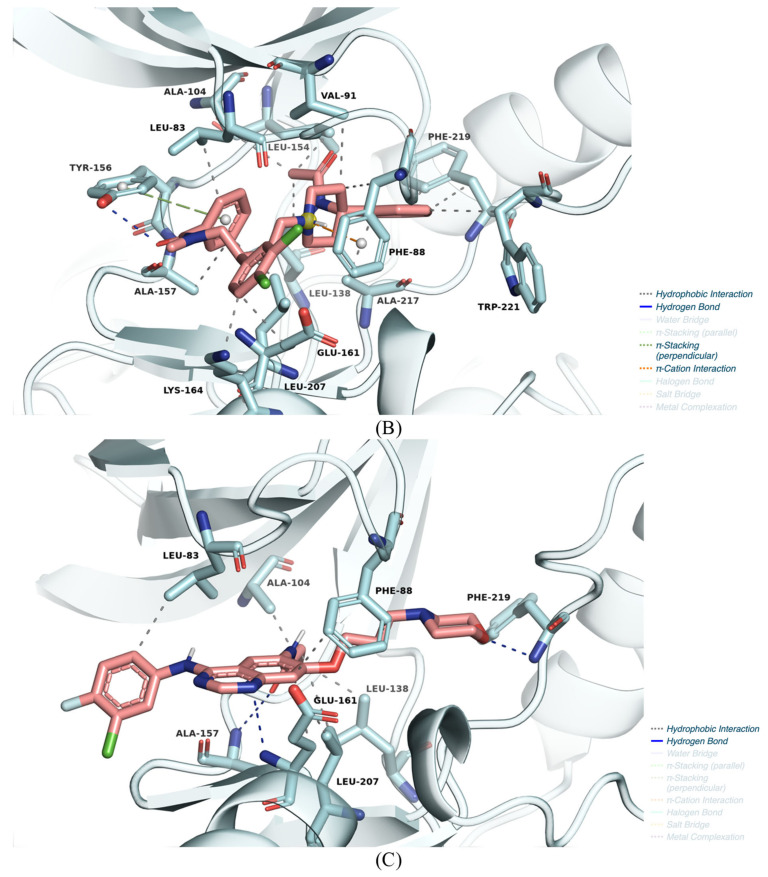
Predicted binding poses and molecular interactions for the repurposed ligands. (**A**) Montelukast; (**B**) saredutant; (**C**) canertinib.

Figure 8A–C illustrates the predicted binding poses and molecular interactions of the three selected candidates within the AurB kinase active site. Hydrophobic interactions dominate the binding mode of montelukast (Figure 8A), with significant contacts involving Leu83, Pro82, and Phe88, contributing to ligand stabilization. Additionally, a hydrogen bond with Lys85 strengthens the interaction, which is further supported by the salt bridge formed with Glu161. The overall binding energy of montelukast (−11.10 kcal/mol) reflects a strong affinity, yet the lack of an MCR scaffold indicates that its mechanism of action may be unconventional, relying more on its interaction network than conserved pharmacophores.

Hydrophobic contacts with residues Leu83, Tyr156, and Ala157 of saredutant (Figure 8B) ensure firm anchoring within the pocket. The hydrogen bond interaction with Glu161, a key residue, highlights its potential for kinase inhibition. Notably, despite the strong binding energy of saredutant (−11.40 kcal/mol) and moderate predicted probabilities in some models, it also lacks a scaffold from one of the established relevant MCRs, suggesting it may act through atypical binding dynamics, making it an interesting candidate for further exploration.

Figure 8C reveals that canertinib engages extensively with hydrophobic residues such as Leu138, Ala157, and Phe88, similar to known inhibitors (e.g., VX-680). Hydrogen bonding with Glu161, Leu138, and Phe219 further stabilizes the binding, while the quinazoline scaffold aligns well within the pocket, reminiscent of other kinase inhibitors. With a binding energy of −10.39 kcal/mol and a high predicted meta-model probability of 99.63, canertinib emerges as a strong candidate for repurposing, especially due to its preserved MCR scaffold.

Given their strong binding energies and favorable interaction profiles within the active site, the three candidates demonstrated significant potential as AurB inhibitors. The compounds were subjected to further validation through molecular dynamics simulations aimed at assessing the stability of the predicted complex with AurB.

### 2.8. Molecular Dynamics Results

**Figure 9:**
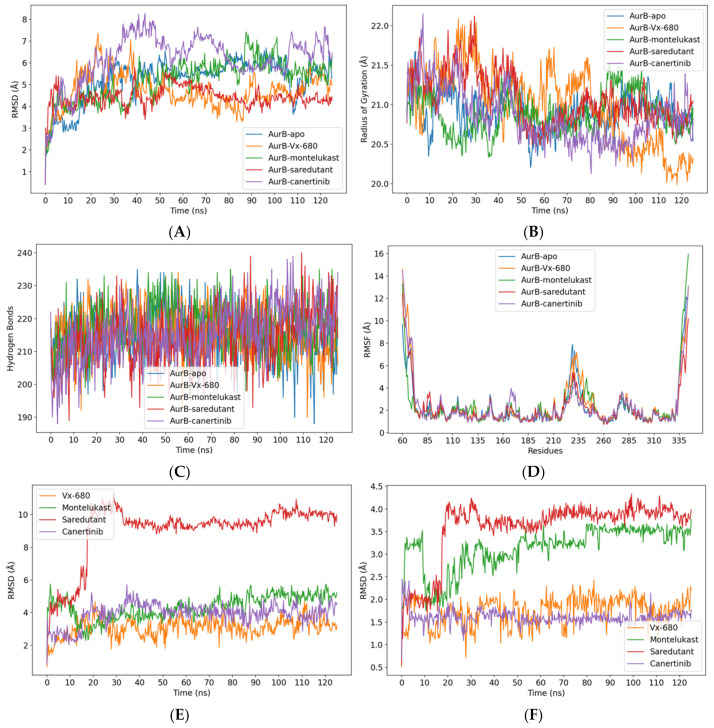
MD results after 125 ns simulation time. (**A**) RMSD values of Cα atoms; (**B**) radius of gyration values; (**C**) total number of intramolecular hydrogen bonds; (**D**) RMSF values for all protein residues; (**E**) RMSD values for ligand movement within the binding site; (**F**) RMSD values for ligand conformations after superposition on initial snapshot.

The MD simulations were conducted to evaluate the stability and dynamic behavior of the AurB–ligand complexes over a 125 ns trajectory. Two reference systems were included: the apo structure of AurB (negative control) and the co-crystallized inhibitor-bound structure (positive control). The results, illustrated in Figure 9, highlighted key differences in the stability and flexibility of the complexes, providing insights into the suitability of the three repurposing candidates.

The root mean square deviation (RMSD) of Cα atoms (Figure 9A) revealed that the ligand-free (apo) structure exhibited higher stability than the complexes with montelukast and canertinib, with RMSD values fluctuating significantly throughout the simulation. Conversely, the co-crystallized inhibitor (VX-680) and the complex with saredutant showed relatively stable trajectories. Among the candidates, the saredutant demonstrated the most stable complex formation, maintaining low RMSD values close to VX-680, indicating a robust binding within the active site.

The radius of gyration (Rg) values (Figure 9B) further supported the structural compactness of the protein–ligand complexes. The Rg values for the apo form fluctuated prominently, suggesting structural instability. The complex with VX-680 maintained a more compact conformation toward the end of the simulation. The complexes with the three candidates showed similar compactness in the last 15 ns., while the AurB–saredutant complex exhibited similar behavior with the positive control in the first 50 ns.

The average number of intramolecular hydrogen bonds (Figure 9C) had similar values for all the simulated systems (214–217). Towards the end of the simulation, a higher number of intramolecular hydrogen bonds was observed for the complexes with saredutant and canertinib.

The root mean square fluctuation (RMSF) per residue (Figure 9D) highlighted localized flexibility across the protein structure. Interestingly, residues in the apo system displayed similar fluctuations with the AurB–VX-680 complex. However, the complexes with the three candidates showed lower fluctuations of the residues within the loop (hinge region) connecting the two lobes. Moreover, some of the residues involved in saredutant binding exhibited higher fluctuations than both the apo structure and other complexes, such as Phe88, which is involved in ATP-binding, illustrating that the initial conformation of the AurB–saredutant complex could represent an intermediate binding mode.

The ligand movement RMSD values (Figure 9E) further revealed that the positive control showed the lowest movement within the binding site during the simulation, followed by canertinib and montelukast. Interestingly, the saredutant showed high movement in the first 25 ns simulation time, which was then stabilized throughout the rest of the trajectory. Ligand conformation RMSD values (Figure 9F) emphasized minimal fluctuations for canertinib within the active site, while VX-680 showed higher conformational changes. Nonetheless, both saredutant and montelukast displayed higher variations in binding conformations. Both ligand movement and conformation RMSD profiles of saredutant illustrated a 25 ns equilibration time of the protein–ligand complex, the fluctuations in RMSD being much lower during the rest of the simulation (last 100 ns). Therefore, it can be assumed that the saredutant underwent a high conformational change in the equilibration phase to engage in more stable binding with the active site. Moreover, the overall ligand movement and ligand conformation RMSD profiles of the last 100 ns showed relatively fewer fluctuations when compared to VX-680. The reference ligand VX-680 had the lowest value for the minimum free binding energy calculated with the MM/PBSA approach (−86.542 kcal/mol), followed by montelukast (−72.098 kcal/mol), saredutant (−65.691 kcal/mol), and canertinib (−55.996), respectively. Binding free energy calculations were performed only for the last 100 ns, corresponding to the production phase.

## 3. Discussion

The developed drug repurposing framework integrated ligand-based and structure-based computational methods to enhance the prediction accuracy of AurB inhibitors. Initially, ligand-based techniques, including scaffold analysis, QSAR modeling, and classification models using molecular fingerprints, were utilized to identify structural features predictive of AurB activity. These approaches informed the creation of a predictive meta-model that combined outputs from QSAR predictions, classification probabilities, and scaffold information to refine candidate selection. This ligand-based meta-model filtered 548 compounds with over 50% probability of inhibitory activity before subjecting them to molecular docking screening. Subsequently, docking-derived interaction fingerprints and binding energies were used to train the final multi-layer perceptron model. This step incorporated molecular interaction data into the prediction process, extending beyond simple quantitative evaluations. By integrating these complementary methodologies, the proposed workflow achieved a balance between structural specificity and prediction robustness, underscoring the synergistic potential of combining ligand- and structure-based approaches in drug discovery.

The structure–activity analysis of the AurB inhibitors provided valuable insights into the chemical features that are important for inhibitory activity. The proposed strategy emphasizes drug repurposing due to its potential to significantly shorten the timeline for bringing new therapies to market. Since repurposed drugs have already undergone extensive clinical testing, including safety and pharmacokinetic evaluations, they can move more swiftly through the regulatory process compared to novel compounds that require comprehensive preclinical and clinical studies.

Other in silico studies on AurB inhibitors have also identified critical binding site residues and employed advanced docking protocols. For example, a study by Sarvagalla et al. highlighted residues Arg159, Glu161, and Lys164 as critical for designing subtype-selective inhibitors due to their solvent-exposed location [30]. Similarly, our docking results align with these findings, particularly highlighting interactions with Glu161 across our proposed candidates. This consistency with previous studies underscores the robustness of our combined framework. Furthermore, the integration of meta-model predictions in our pipeline extends the capabilities of traditional QSAR or docking-only methods, a step forward compared to other similar works in the literature [20,25,26].

This study demonstrates the powerful synergy of computational tools in drug discovery. By integrating docking simulations with machine learning models, we developed a comprehensive framework to predict AurB inhibitors, identifying saredutant, montelukast, and canertinib as promising candidates. Molecular dynamics (MD) simulations revealed that saredutant could potentially be the most suitable candidate for repurposing. The initial fluctuations (first 25 ns) of the saredutant may represent conformational adjustments as the ligand better accommodates into the binding pocket, showing more stable dynamics in the following 100 ns. However, the complexity of the predictive models using advanced machine learning techniques hinders interpretability, making it difficult to clearly understand the molecular features that contribute mostly to activity. Another limitation of our study is the nature of the molecular descriptors used in QSAR modeling since 6 out of 10 features represent sums of van der Waals surface area contributions of specific atoms based on several ranges for partial charges, lipophilicity, molecular refractivity, and electrotopological state indices, while three of the features combine van der Waals surface areas and different bins of atomic partial charges computed using PEOE method.

Despite these promising findings, experimental validation remains essential to confirm the inhibitory activity of the identified compounds. The achievement of this study lies not only in the identification of the potential repurposed drugs but also in the development of a versatile, integrated computational pipeline for drug discovery. This combined method, which incorporates docking, machine learning, and molecular dynamics, is broadly applicable and can be adapted to other drug targets beyond AurB. Additionally, leveraging structural insights gained from this study can orient de novo drug design, offering a pathway to novel inhibitors with greater specificity and improved therapeutic profiles.

## 4. Materials and Methods

**Figure 10:**
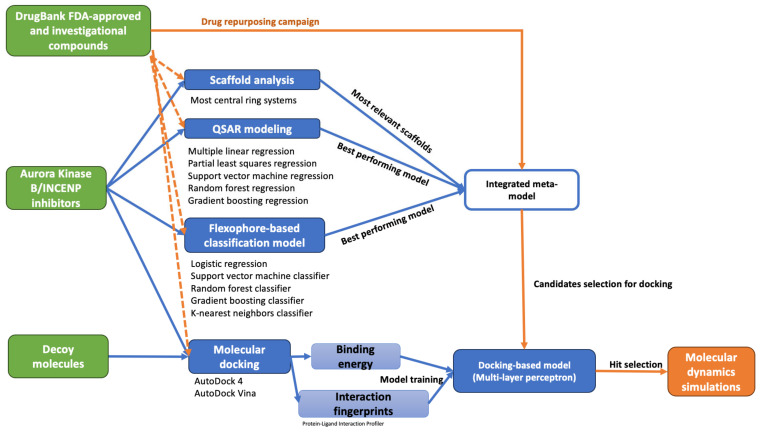
Workflow diagram illustrating the methodology implemented for the discovery of novel AurB inhibitors through drug repurposing approaches.

A drug repurposing framework was developed by stacking both ligand-based and structure-based computer-aided drug discovery methods aiming at identifying novel potential AurB inhibitors among FDA-approved and investigational drugs. ML-aided approaches were implemented at each step of the workflow. The developed drug repurposing framework is schematically illustrated in Figure 10.

### 4.1. Dataset Preparation

SMILES codes of 192 AurB/INCENP inhibitors (CHEMBL3430907) and their pharmacological potency values (IC50) were retrieved from the ChEMBL database. We chose this particular dataset with a low number of observations in order to have a high homogeneity between the experimental assays. Duplicate entries were merged, and multiple potency values were expressed as the arithmetic mean. The retrieved virtual library was divided into two datasets, one containing only the compounds with exact IC50 values and the other retaining the compounds with inexact or missing potencies, labeled as inactive. Activities were thereafter expressed as their negative logarithmic values (pIC50).

A decoy library was also prepared for further molecular docking studies. Using DataWarrior v06.03.01 software [29], the retrieved AurB/INCENP inhibitors were clustered by Tanimoto similarity indices into 50 clusters, and 50 representative compounds (1 per cluster) were selected for decoys retrieval. Thereafter, the DUD-E server was used to generate a total of 550 chemically diverse decoy structures [31].

For the repurposing study, the DrugBank database was retrieved along with the three-dimensional (3D) coordinates of the included compounds. The library was then filtered to retain only FDA-approved drugs or compounds involved in clinical trials, thus being in an advanced development stage (investigational drugs).

### 4.2. Scaffold Analysis of AurB Inhibitors

The AurB library containing only the compounds with exact pIC50 values was used to generate MCR fragments using DataWarrior v06.03.01 software. Compounds were divided into two subsets as follows: potent inhibitors (PI) with pIC50 values of at least 6.5 M and weak inhibitors (WI) with pIC50 < 6.5 M. We established a cutoff value of pIC50 = 6.5 M (IC50 of approx. 317) to divide the dataset into relatively balanced subsets, while also considering this potency threshold suitable enough to discriminate between strong and weak binders. Thereafter, AurB inhibitors were grouped by their corresponding MCRs for statistical analysis. After calculating the mean pIC50 values and the proportion of potent compounds for each MCR group, we performed non-parametric Mann-Whitney U tests to compare the pIC50 distributions of each MCR group against all other MCRs combined. Using the resulting p values, we determined which scaffold is characteristic of inhibitors with statistically significant higher potency. Furthermore, for the same rationale, we performed Chi-square tests for the proportions of compounds in the potent subset, and the standardized residuals were also computed to identify specific scaffolds with significant deviations (residuals over ± 1.96 were considered significant at the 95% confidence interval level) [32,33,34].

The EF values for each MCR scaffold were calculated as the ratio between the frequency in the potent subset and the overall frequency in the dataset, determining which particular scaffold is enriched for potent compounds relative to the entire AurB inhibitors. Lastly, MCR scaffolds with enrichment factors > 1 and statistically higher potencies were labeled accordingly and were transformed by one-hot encoding in binary features for further use in machine learning (compounds that contained the relevant MCR were labeled as 1 for that specific scaffold, they were labeled as 0).

### 4.3. QSAR Modeling

Several quantitative structure-activity relationship (QSAR) models were built using the library containing exact potency values for AurB inhibitors. All operations were performed in Python 3.11 programming language.

#### 4.3.1. Feature Selection

Firstly, 208 molecular descriptors (constitutional, topological, electronic, geometric, and hybrid descriptors) were generated using RDKit v2024.09.2 [35]. Preprocessing of descriptors included the removal of columns with non-numerical and missing values. Feature selection techniques were employed to select the most suitable molecular descriptors (independent variables), as follows: a variance threshold of 0.01 was applied to remove descriptors with low variance, the absolute correlation matrix was determined to eliminate variables with a correlation coefficient > 0.85 with any other descriptor to mitigate multicollinearity, and a random forest regressor was employed for recursive feature elimination (RFE) on the remaining variables to select the top 10 most significant descriptors. The remaining descriptors were thereafter standardized prior to model training.

#### 4.3.2. Model Training and Validation

Five predictive models were trained to estimate the pIC50 values based on selected molecular descriptors. The dataset was split into training and test sets with an 8:2 ratio. Multiple linear regression (MLR), partial least squares regression (PLS), support vector machine regression (SVM), random forest regression (RF), and gradient boosting regression (GB) models were developed using scikit-learn 1.5.2 Python library (https://scikit-learn.org/stable/about.html, accessed on 3 October 2024) with standard hyperparameters. The performance of each model was assessed by performing five-fold cross-validation on the training set and by calculating several metrics for both training and test sets, including R^2^ (determination coefficient), RMSE (root mean square error), MAE (mean absolute error), and Q^2^ (cross-validated R2). Based on the specific metrics, the best-performing model was selected for further analysis.

Various explainable ML techniques were applied to enhance the interpretability of the best-performing model. Thus, permutation importance was computed to quantify the contribution of each feature to the predictions, with a higher decrease in performance indicating a higher importance. Moreover, individual conditional expectation (ICE) and partial dependence plots (PDP) were generated to visualize the influence of each descriptor on the predicted biological activity. Lastly, Shapley Additive explanations (SHAP) analysis was performed to estimate the contribution of each feature to the model predictions on the training set using the SHAP library in Python [36].

#### 4.3.3. Applicability Domain Analysis

Three distinct methods were employed to determine the applicability domain of the best-performing model. Therefore, a leverage-based approach was used to generate a William plot based on leverage values and studentized residuals for both the training and test sets. Furthermore, a PCA was performed to capture the variation in the descriptor space, using the first two principal components to represent the compound distribution in a reduced-dimensionality space, assessing the relative location of the test set compounds compared to the training set. A feature range analysis was also performed by determining the minimum and maximum values of the selected molecular descriptors, ensuring that the test set compounds fall within the chemical space covered by the training set.

### 4.4. Flexophore-Based Classification Models

We further built several classification models using flexophore fingerprints as independent variables to predict whether a compound is active or inactive toward AurB. Flexophores are 3D molecular descriptors that capture both the conformational flexibility and pharmacophoric features (hydrogen bond donors/acceptors, aromatic centers, etc.) of molecules [34,37]. The AurB/INCENP inhibitors library with both exact and inexact/missing pIC50 values was used and was split into two activity classes based on a biological activity threshold, established to also maintain class balance: active (pIC50 ≥ 6 M, y = 1) and inactive (pIC50 < 6 or missing, y = 0) compounds. A cutoff value of pIC50 = 6 M (IC50 = 1000 nM) was established in order to maintain class balance, with compounds with IC50 values in the submicromolar range being labeled as actives. Even if compounds with low activity values are still known to bind to AurB at high concentrations, we chose to label them as “inactive” together with structures with missing potency data as a consensus to maintain consistency with the binary architecture of the classification models. All data processing operations and model training and evaluation were performed in Python 3.11 programming language.

#### 4.4.1. Data Processing

Flexophore fingerprints were generated using DataWarrior software and exported as strings, which were thereafter used to construct the feature matrix. A dictionary was used to count the occurrences of each pattern for each compound entry, while the frequency count for each pattern was also added to the feature matrix. Therefore, a structured feature set was created where each feature represents the occurrence of a unique flexophore pattern for each compound.

Furthermore, a multi-step feature processing pipeline was implemented to prepare the independent variables for training classification models using the scikit-learn library. First, a variance threshold was applied to remove features with variance < 0.01. Secondly, the remaining features were standardized using standardscaler to ensure each feature has a mean of 0 and a standard deviation of 1. Lastly, a principal component analysis (PCA) was applied to reduce feature dimensionality, establishing the transformation to retain the top 20 principal components, capturing most of the flexophore variability.

#### 4.4.2. Training and Validation

The created dataset was split into training and test sets at an 8:2 ratio using standard hyperparameters and 10 different random seeds to create a stratified sampling. Classification models were trained using each different seed to assess the stability and robustness of each model, maintain class distribution, and identify the model with the best overall performance. A five-fold cross-validation was also performed on the training set for each seed and model. Five different classification models were trained on each partitioning seed using standard hyperparameters, including logistic regressions (LR), support vector machines (SVM), random forest classifiers (RF), gradient boosting classifiers (GB), and K-nearest neighbors classifiers (KNN). The performance of each model was evaluated by computing the following metrics for training, cross-validations, and test sets: accuracy, balanced accuracy (bACC, average of sensitivity and specificity), sensitivity (recall, true positive rate), specificity (true negative rate), F1 score (harmonic mean of precision and recall), ROC AUC (area under receiver operating characteristic curve), and Matthews correlation coefficient (MCC, a measure of the quality of binary classifications). The best-performing model was selected for further use on the repurposing dataset.

### 4.5. Integrated Ligand-Based Meta-Model Training

A stratified logistic regression model was developed to combine the presence of the most relevant central rings specific to potent AurB inhibitors with the output of previously trained regression and classification models. Therefore, previously established activity classes (0 or 1) of the complete AurB inhibitors dataset were used as dependent variables, while the predicted pIC50 values by the selected QSAR model, predicted probabilities by selected flexophore-based classification model, and the one-hat encoded vectors for MCR scaffolds were used as independent variables (two continuous features and three binary features). Moreover, both predicted pIC50 values and probabilities were standardized from −1 to 1. Logistic regression with L2 regularization (ridge regression) with default strength was chosen as a meta-model due to its simplicity and high interpretability. Training and evaluation of the logistic regression models were performed similarly to Section 4.2, using 10 different random seeds for dataset splitting. The selected meta-model was thereafter used to select candidates for molecular docking among the repurposing dataset.

### 4.6. Structure-Based Machine Learning

Molecular docking studies were performed on both the AurB inhibitors set and the generated decoy molecules to predict binding energies and extract interaction fingerprints for further modeling. To this extent, both AutoDock 4 (AD4) [38] and AutoDock Vina 1.1.2 [39] were used to identify the best-performing algorithm for predicting AurB binders.

#### 4.6.1. Protein and Ligand Structures Preparation

The crystal structure of the AurB/INCENP complex bound to the VX-680 inhibitor was retrieved from the RCSB PDB database (PDB: 4AF3, 2.75 Å resolution) [40]. Water molecules were removed, and the residues were protonated at physiological pH (7.4), also optimizing the H-bond network. The missing loop was modeled using YASARA Structure [41], and the full structure was optimized by performing energy minimization with AMBER14 forcefield and a short molecular dynamics (MD) simulation of 100 ps in explicit solvent.

Ligands for docking were prepared by generating 3D structures of AurB inhibitors and retrieved decoys by energy minimizations with MMFF94s+ forcefield using DataWarrior and by protonation at physiological pH [29].

#### 4.6.2. Molecular Docking Simulations

Molecular docking studies were carried out by setting the grid box to include only the kinase active site. Simulations were performed using both AD4 and Vina with 20 runs for each compound. The accuracy and performance of each method were assessed by multiple approaches. Firstly, the co-crystallized ligand was re-docked into the binding site, and the RMSD was calculated after superposition during the initial conformation. Furthermore, ROC (receiver operating characteristic) curves were generated, and ROC AUC values were calculated using the predicted binding energies to assess the capacity of the two algorithms to discriminate quantitatively between actives and inactives/decoys. Enrichment was also performed for ROC analysis by calculating logarithmic values for false positive rates and for AUC (logAUC). Mann–Whitney U tests were also performed to analyze the statistical significance of differences in binding energies between active and inactive molecules for each docking method [42]. Binding poses and molecular interactions were depicted using PyMOL v3.0.4 (The PyMOL Molecular Graphics System, Schrödinger, LLC, New York, NY, USA) and Protein-Ligand Interaction Profiler software (PLIP) [43].

### 4.7. Post-Docking Analysis

#### 4.7.1. Interaction Fingerprints Extraction and Analysis

Protein–ligand interactions predicted with both docking algorithms were generated using PLIP [43]. The protocol was applied for both the screened AurB inhibitors, decoys, and the co-crystallized ligand. Reports of each interaction type were used to construct vectors of binary fingerprints. For each ligand, the generated features represented the presence (1) or absence (0) of a specific interaction with a particular residue within the binding site.

To qualitatively assess the reliability of the docking algorithms in predicting relevant interactions with specific residues, Tanimoto similarity indices (the ratio between the number of features shared between two compounds and the total number of unique features across both compounds) using Jaccard distance metric were computed between the fingerprints generated for active molecules and the co-crystallized ligand, as a reference fingerprint. The docking method, which yielded the highest mean Tanimoto similarity value and the best separation between active and inactive ligands, was selected for further studies.

#### 4.7.2. Multi-Layer Perceptron Model

A classification model was developed based on the results provided by the best-performing docking algorithm, using normalized predicted binding energies and binary interaction fingerprints. The predicted interactions with the highest relevance for inhibitory activity were selected using two methods. Chi-square tests were performed to assess the dependence between each interaction feature and ligand activity, while a random forest classifier was used to compute feature importance scores for each interaction. With each method, the top 10 features were retained, and the identified predicted interactions that were common for both approaches were selected as features for model training.

Furthermore, AurB inhibitors with pIC50 ≥ 6 M were categorized as active ligands, while decoys were merged with low-activity inhibitors and formed the inactive ligands set; the whole dataset is split into training and test sets (8:2 ratio). The cutoff value for splitting the dataset into two activity classes was established in order to maintain consistency with the previously trained flexophore-based classification models and the logistic regression meta-model. Using standardized binding energies and selected binary interaction fingerprints, multi-layer perceptron (MLP) neural networks were trained with keras library by fine-tuning hyperparameters such as learning rate (0.001, 0.01, 0.05, 0.1), batch size (8, 16, 32, 64) and dropout rate (0.1, 0.2, 0.3, 0.4). The network architecture consisted of two hidden layers with 32 and 16 neurons, respectively, and a RelU activation function, while the output layer had a sigmoid activation function. Furthermore, a moderate noise of 0.01 was added to binding energy to reduce its influence on predictions, while a class weight scaling factor was computed for active class (1) to address the class imbalance. A 10-fold cross-validation was applied for each hyperparameter combination to identify the most accurate model. The performance of the selected model was further evaluated by calculating the same performance metrics as in Section 4.2. Feature importance values for the best-performing model were calculated by analyzing the weights of the first dense layer in the neural network.

### 4.8. Drug Repurposing Study

Structures of approved and investigational drugs retrieved from DrugBank (repurposing compounds) were subjected to all data processing steps described for AurB inhibitors and decoys. Thereafter, a filtering approach was performed to select only the DrugBank entries that fall within the applicability domain of the selected QSAR regression and flexophore-based classification model. For the latter case, drug structures were considered within the applicability domain if they shared with compounds used for model training a flexophore similarity index greater than 0.8. After retaining the molecules that passed the applicability domain check, pharmacological potency (pIC50) values and probabilities of being active were predicted using the previously selected QSAR and classification models. The yielded outputs, along with the generated MCR binary vectors, were then used with the logistic regression meta-model. Drug molecules predicted as active with the meta-model were thereafter subjected to molecular docking studies using the most suitable docking algorithm. Estimated binding energies and interaction fingerprints were further used for predictions based on the fine-tuned MLP model. Of the top-ranked drugs with probabilities of being true binders, over 95% were selected as repurposing candidates for MD simulations [33,34].

### 4.9. Molecular Dynamics Simulations

MD simulations were performed for the selected repurposing candidates to evaluate the stability of the predicted AurB–candidate complexes over time. Two reference systems were also included in the simulations: the ligand-free (apo) structure as a negative control and the complex with the co-crystallized inhibitor as a positive control. Simulations were conducted using YASARA Structure for a total duration of 100 ns [41]. The simulated systems were neutralized by adding NaCl ions to achieve 0.9% concentration; energy minimizations were performed using the steepest descent and simulated annealing approaches to remove potential steric clashes. The following force fields were applied: AMBER14 for protein structures, GAFF2 and AM1BCC for ligand atoms, and TIP3P for water molecules. Van der Waals interactions were computed with an 8 Å cutoff, while long-range electrostatic interactions were calculated using the particle mesh Ewald method without a cutoff. Simulations were maintained at 298 K and 1 atm, corresponding to the NPT ensemble. The equations of motion were integrated with a multiple timestep approach of 5 fps for non-bonded and 2.5 fs for bonded interactions. Free energies of binding (kcal/mol) were estimated using the Poisson–Boltzmann (MM/PBSA) method, excluding the entropic contribution [44].

## 5. Conclusions

The integrated computational pipeline developed in this study identified promising repurposing candidates for AurB inhibition, also demonstrating robust performance across multiple predictive and validation steps. By leveraging scaffold-specific information alongside QSAR and classification outputs, this approach provides a comprehensive framework for identifying potent inhibitors within diverse chemical spaces. Beyond these findings, the broader significance lies in the versatility of our methodology, which can be adapted to other challenging targets in drug discovery. These results pave the way for experimental validation and refinement, advancing both our understanding of AurB inhibition and computational approaches in pharmacology.

## Figures and Tables

**Table 1:** Definitions of molecular descriptors used for QSAR modeling and the ranges specific to the training set (applicability domain) (n = 127).

Molecular Descriptor	Minimum Value	Maximum Value	Definition
qed	0.149	0.831	quantitative estimate of drug-likeness, integrating multiple physicochemical properties into a single score
SPS	10.063	25.975	Special score, an empirical metric which quantifies the spatial complexity
BCUT2D_CHGLO	−2.489	−1.972	Lowest eigenvalues of the Burden matrix, with diagonal elements weighted by atomic partial charges
PEOE_VSA2	4.682	19.672	Total van der Waals surface area of atoms with partial charges (−0.30, −0.25) calculated using the PEOE method
PEOE_VSA7	17.696	96.456	Total van der Waals surface area of atoms with partial charges (−0.05, 0.00) calculated using the PEOE method
PEOE_VSA8	11.760	73.553	Total van der Waals surface area of atoms with partial charges (0.00, 0.05) calculated using the PEOE method
SMR_VSA10	0.000	63.219	Total van der Waals surface area of atoms with individual contributions to molar refractivity over 4
SlogP_VSA11	0.000	11.760	Total van der Waals surface area of atoms with individual contributions to SlogP between 0.5 and 0.6
EState_VSA8	4.984	88.878	Total van der Waals surface area of atoms with individual Estate (electrotopological state) indices between 2.05 and 4.69
VSA_EState3	0.000	25.082	Sum of EState indices for atoms with individual van der Waals surface area contribution between 5 and 5.41 Å

**Table 2:** Performance metrics for the five trained regression models.

	Train R^2^	Test R^2^	Train RMSE	Test RMSE	Train MAE	Test MAE	Q^2^
MLR	0.5551	0.4239	0.7472	0.8712	0.5924	0.6904	0.3403 ± 0.0992
PLS	0.5489	0.3896	0.7524	0.8967	0.5908	0.7245	0.3268 ± 0.1054
SVM	0.9764	0.8054	0.1722	0.5063	0.1332	0.3949	0.6697 ± 0.0985
RF	0.9587	0.7623	0.2275	0.5595	0.1734	0.4177	0.6574 ± 0.0932
GB	0.9949	0.7580	0.0800	0.5646	0.0484	0.3987	0.6099 ± 0.1414

## Data Availability

The original contributions presented in the study are included in the article/Supplementary Material, further inquiries can be directed to the corresponding authors.

## Supplementary Materials

The following supporting information can be downloaded at https://www.mdpi.com/article/10.3390/ph18010013/s1, Table S1. Descriptive statistics for the dataset of AurB inhibitors; Table S2. Descriptive statistics for the dataset of decoy compounds generated with DUD-E platform; Table S3. Mean potency values and frequency within potent AurB inhibitors for the generated most central ring (MCR) systems; Figure S1. Correlation matrix for the selected molecular descriptors for QSAR modeling and experimental pIC50 values; Figure S2. Correlation diagrams between experimental and predicted pIC50 values for the five trained models; Figure S3. Partial dependence (PD) and individual conditional expectation (ICE) plots for the selected molecular descriptors; Figure S4. Analysis of applicability domain for the selected SVM regression model; Table S4. Range values for the selected molecular descriptors; Table S5. Performance metrics for the training set, test set, and five-fold cross-validation; Table S4. Performance metrics for the trained logistic regression stacking model; Figure S5. Superposition of binding poses predicted with Vina; Figure S6. Correlation between docking results and experimental potency and efficiency values; Figure S7. Selected interactions for model building; Table S5. Performance metrics for the trained MLP model based on binding energies and interaction fingerprints; Table S6. The top 30 repurposable compounds were ranked by predicted probabilities estimated using the docking-based MLP model.
