# Supplementary material for: Machine Learning-Assisted Drug Repurposing Framework for Discovery of Aurora Kinase B Inhibitors
Source: Pharmaceuticals (Basel). 2024 Dec 25;18(1):13. doi: 10.3390/ph18010013 (PMC11768374; doi:10.3390/ph18010013)
Supplement: Supplementary file 1 [file pharmaceuticals-18-00013-s001.zip › pharmaceuticals-3385089-supplementary.pdf]

Supplementary Material

Results

2.1. Dataset preparation

Table S1. Descriptive statistics for the dataset of AurB inhibitors (Total Dataset, n=179 and EV Set, n=127)

Descriptor	Dataset	Minimum	Maximum	Mean	Standard Deviation	Variance
**Molecular Weight (g/mol)**	Total Dataset	184.2	663.5	~450.0	±74.8	5597.1
	EV Set	198.2	663.5	~470.0	±71.2	5069.0
**AlogP**	Total Dataset	1.96	6.94	~4.8	±1.1	1.3
	EV Set	2.24	6.94	~4.9	±1.0	1.0
**pIC50**	Total Dataset	N/A	N/A	N/A	N/A	N/A
	EV Set	4.0	9.3	~6.4	±0.8	0.64
**H-Acceptors**	Total Dataset	2	11	~6.5	±1.5	2.3
	EV Set	3	11	~6.7	±1.4	2.0
**H-Donors**	Total Dataset	0	4	~2.0	±1.1	1.2
	EV Set	0	4	~2.2	±1.0	1.0
**Relative PSA**	Total Dataset	0.063	0.639	~0.27	±0.08	0.006
	EV Set	0.073	0.639	~0.29	±0.07	0.005
**Molecular Flexibility**	Total Dataset	0.210	0.446	~0.32	±0.07	0.005
	EV Set	0.220	0.437	~0.33	±0.06	0.004
**Molecular Complexity**	Total Dataset	0.75	0.95	~0.85	±0.05	0.003
	EV Set	0.77	0.94	~0.87	±0.04	0.002
**Rotatable Bonds**	Total Dataset	2	10	~6.0	±1.5	2.4
	EV Set	3	9	~6.3	±1.4	2.0
**Small Rings**	Total Dataset	3	7	~5.0	±1.1	1.2
	EV Set	3	7	~5.1	±1.0	1.0

Table S2. Descriptive statistics for the dataset of decoy compounds generated with DUD-E platform (n=550)

Descriptor	Minimum	Maximum	Mean	Standard Deviation	Variance
**Molecular Weight (g/mol)**	113.16	348.87	244.55	56.12	3149.51
**AlogP**	-5.79	6.51	1.41	1.83	3.36
**H-Acceptors**	0	8	2.97	1.47	2.15
**H-Donors**	0	4	1.16	0.93	0.87
**Relative PSA**	0	0.63	0.25	0.11	0.01
**Molecular Flexibility**	0	0.88	0.36	0.18	0.03
**Molecular Complexity**	0.34	0.95	0.7	0.1	0.01

**Rotatable Bonds**	0	10	2.27	1.79	3.21
**Small Rings**	0	6	2.36	1.1	1.21

2.2. Scaffold analysis

Table S3. Mean potency values and frequency within potent AurB inhibitors for the generated most central ring (MCR) systems.

MCR	Frequency within potent inhibitors	Mean pIC50	p (Mann-Whitney)
1+2	1.00	8.15	0.0038
3	0.11	5.68	0.0045
4	0.52	6.28	0.3735
5	0.63	6.73	0.0903
6	0.20	5.06	0.0080
7	0.29	6.23	0.0177
8	0.67	7.86	0.0304
9	1.00	7.57	0.0004

2.3. QSAR modeling

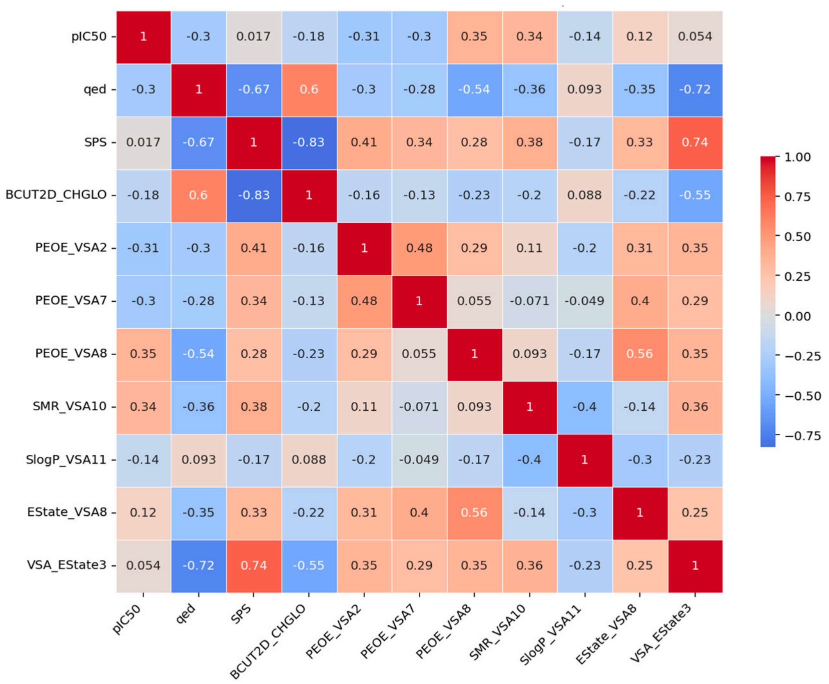

Figure S1. Correlation matrix for the selected molecular descriptors for QSAR modeling and experimental pIC50 values.

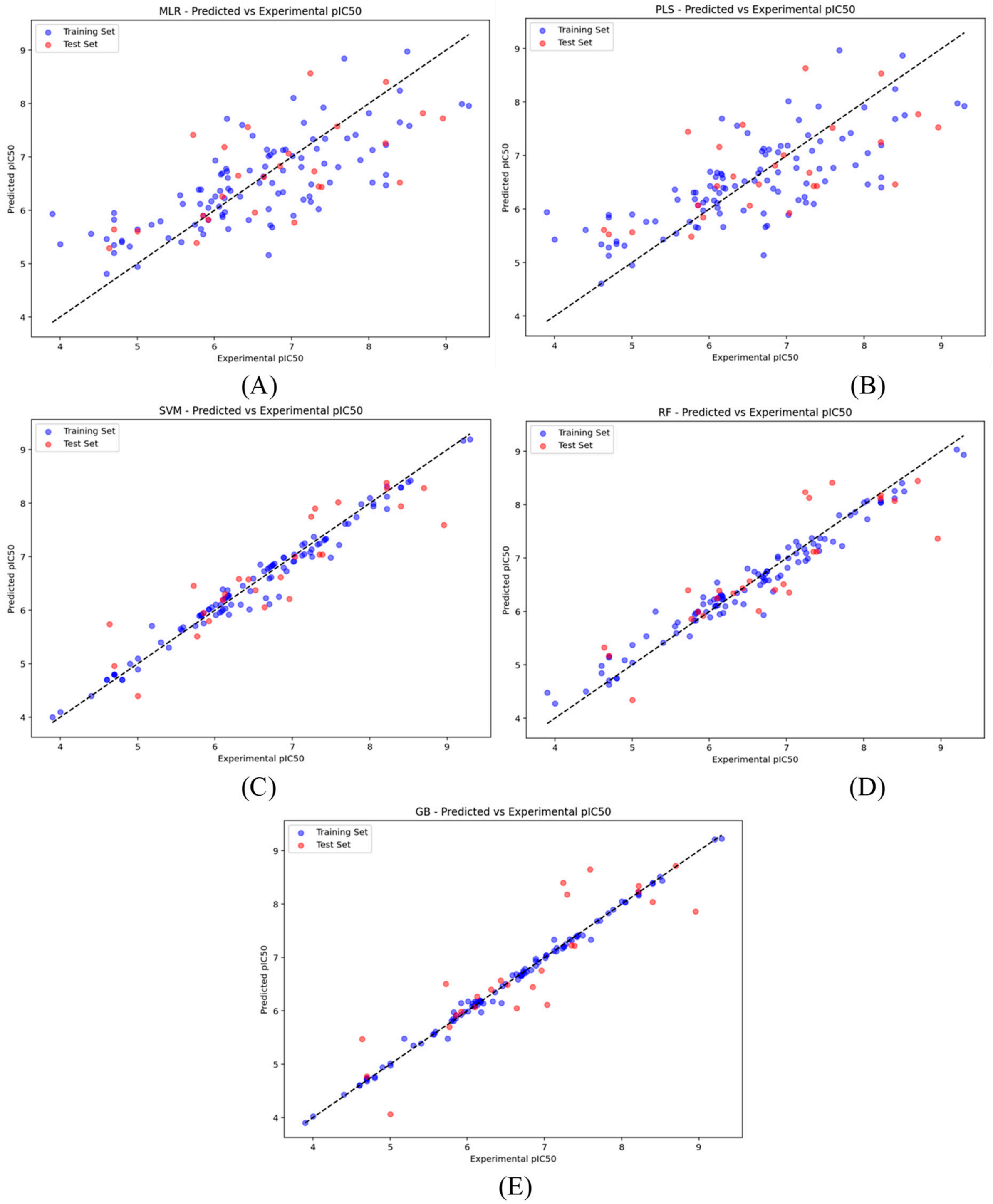

Figure S2. Correlation diagrams between experimental and predicted pIC50 values for the 5 trained models. (A) Multiple linear regression (MLR); (B) partial least squares regression (PLS); (C) support vector machine regression (SVM); (D) random forest regression (RF); (E) gradient boosting regression (GB).

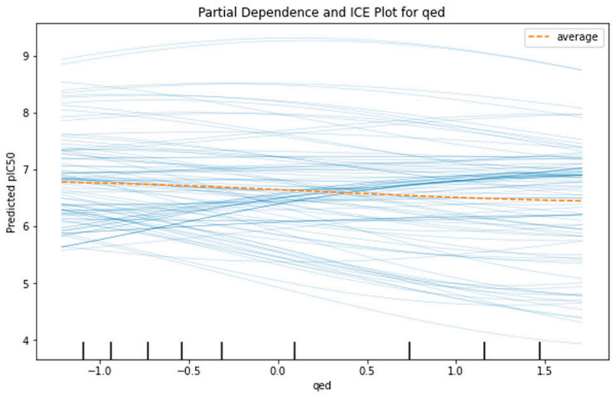
Partial Dependence and ICE Plot for qed
average
Predicted pIC50
qed

(A)

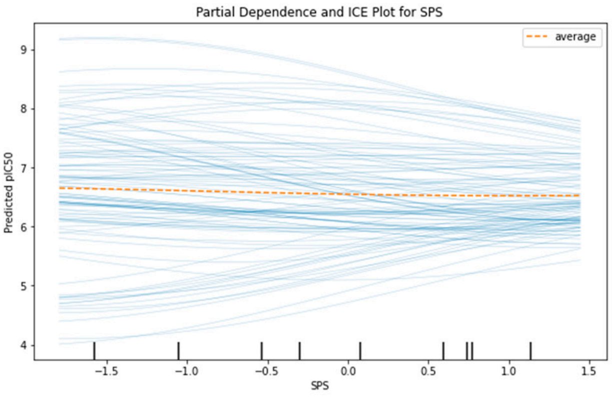
Partial Dependence and ICE Plot for SPS
average
Predicted pIC50
SPS

(B)

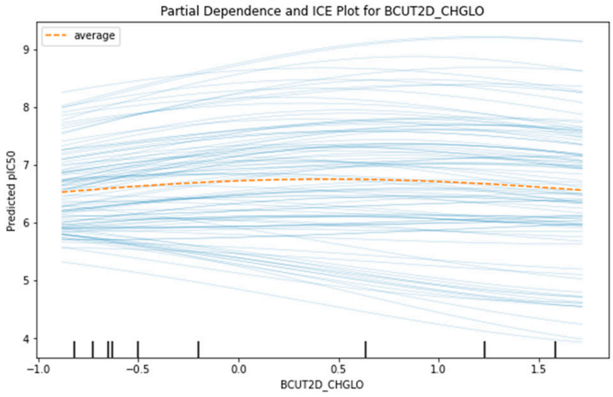
Partial Dependence and ICE Plot for BCUT2D_CHGLO
average
Predicted pIC50
BCUT2D_CHGLO

(C)

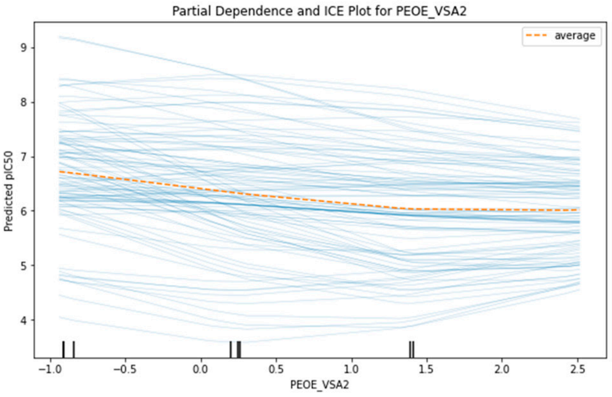
Partial Dependence and ICE Plot for PEOE_VSA2
average
Predicted pIC50
PEOE_VSA2

(D)

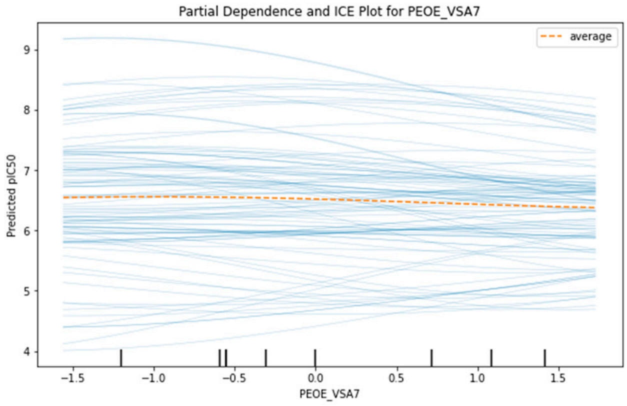
Partial Dependence and ICE Plot for PEOE_VSA7
average
Predicted pIC50
PEOE_VSA7

(E)

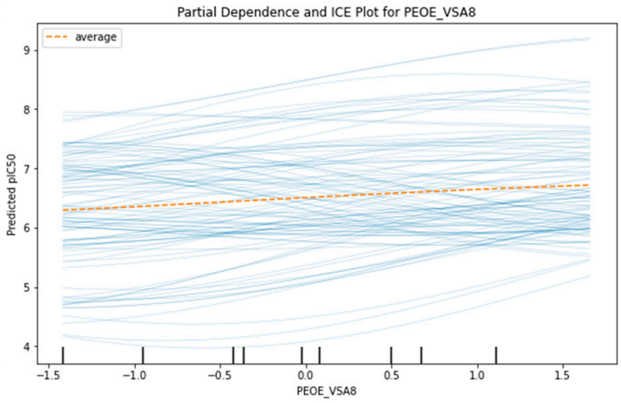
Partial Dependence and ICE Plot for PEOE_VSA8
average
Predicted pIC50
PEOE_VSA8

(F)

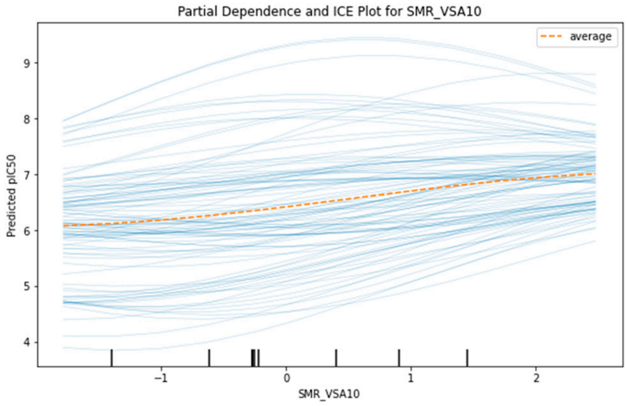
Partial Dependence and ICE Plot for SMR_VSA10
average
Predicted pIC50
SMR_VSA10

(G)

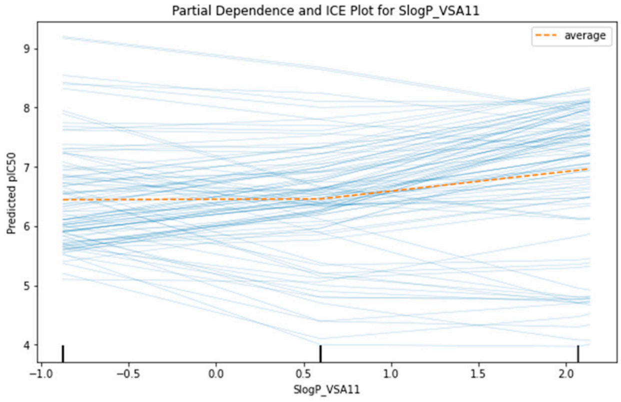
Partial Dependence and ICE Plot for SlogP_VSA11
average
Predicted pIC50
SlogP_VSA11

(H)

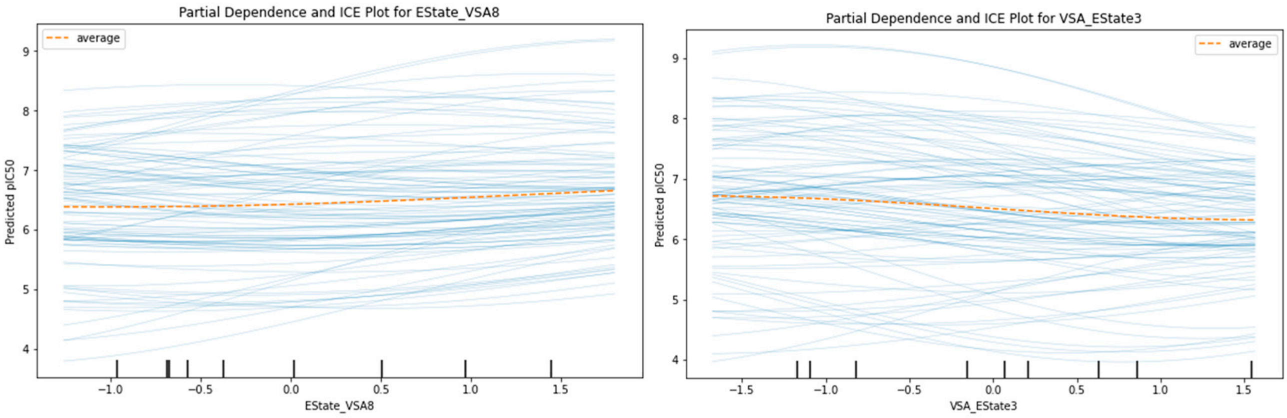

(I) (J)

Figure S3. Partial dependence (PD) and individual conditional expectation (ICE) plots for the selected molecular descriptors. (A) qed; (B) SPS; (C) BCUT2D_CHGLO; (D) PE0E_VSA2; (E) PE0E_VSA7; (F) PE0E_VSA8; (G) SMR_VSA10; (H) SlogP_VSA11; (I) EState_VSA8; (J) VSA_Estate3.

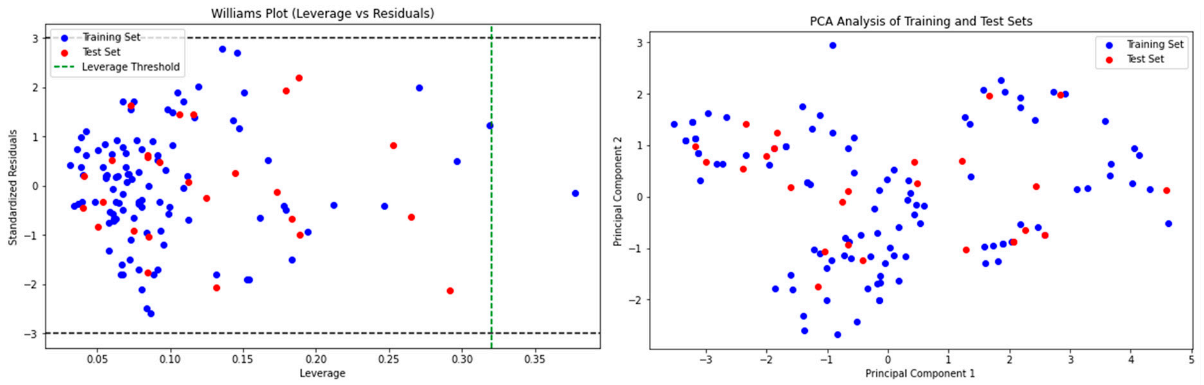

(A) (B)

Figure S4. Analysis of applicability domain for the selected SVM regression model. (A) Williams plot; (B) PCA analysis of feature space.

Table S4. Range values for the selected molecular descriptors.

Descriptor	Min Train	Max Train	Min Test	Max Test
qed	0.15	0.83	0.19	0.87
SPS	10.06	25.98	10.23	25.18
BCUT2D_CHGLO	-2.49	-1.97	-2.48	-2.07
PEOE_VSA2	4.68	19.67	4.68	19.67
PEOE_VSA7	17.70	96.46	35.90	99.52
PEOE_VSA8	11.76	73.55	11.76	61.43
SMR_VSA10	0.00	63.22	0.00	52.29
SlogP_VSA11	0.00	11.76	0.00	11.50
EState_VSA8	4.98	88.88	10.30	64.39
VSA_EState3	0.00	25.08	0.00	22.07

2.4. Fingerprints classification models

Table S5. Performance metrics for the training set, test set and 5-fold cross-validation.

	**Training set**						
Model	**Accuracy**	**bACC**	**Sensitivity**	**Specificity**	**F1 Score**	**ROC AUC**	**MCC**
LR	0.8531	0.8551	0.8333	0.8769	0.8609	0.9421	0.7075
SVM	0.8881	0.8885	0.8846	0.8923	0.8961	0.9710	0.7752
RF	0.9720	0.9718	0.9744	0.9692	0.9744	0.9982	0.9436
GB	0.9720	0.9718	0.9744	0.9692	0.9744	0.9982	0.9436
KNN	0.9231	0.9218	0.9359	0.9077	0.9299	0.9776	0.8448
	**Cross-validation**						
Model	**Accuracy**	**bACC**	**Sensitivity**	**Specificity**	**F1 Score**	**ROC AUC**	**MCC**
LR	0.8027 ± 0.1364	0.8012 ± 0.1203	0.7991 ± 0.2372	0.8034 ± 0.0904	0.7977 ± 0.1723	0.8792 ± 0.1397	0.6066 ± 0.2313
SVM	0.8103 ± 0.0947	0.8088 ± 0.0815	0.8116 ± 0.2093	0.8061 ± 0.0899	0.8071 ± 0.1275	0.8689 ± 0.1431	0.6232 ± 0.1432
RF	0.8313 ± 0.1093	0.8302 ± 0.1075	0.8132 ± 0.1982	0.8472 ± 0.0706	0.8275 ± 0.1410	0.9102 ± 0.0702	0.6697 ± 0.2144
GB	0.7899 ± 0.1188	0.7895 ± 0.1168	0.7741 ± 0.1798	0.8049 ± 0.0748	0.7894 ± 0.1436	0.8893 ± 0.0833	0.5819 ± 0.2323
KNN	0.8042 ± 0.0633	0.8196 ± 0.0334	0.8389 ± 0.1455	0.8004 ± 0.1299	0.8163 ± 0.0653	0.9269 ± 0.0392	0.6375 ± 0.0894
	**Test set**						
Model	**Accuracy**	**bACC**	**Sensitivity**	**Specificity**	**F1 Score**	**ROC AUC**	**MCC**
LR	0.8611	0.8625	0.8500	0.8750	0.8718	0.9594	0.7216
SVM	0.8611	0.8563	0.9000	0.8125	0.8780	0.9094	0.7181
RF	0.9167	0.9188	0.9000	0.9375	0.9231	0.9688	0.8336
GB	0.9444	0.9500	0.9000	1.0000	0.9474	0.9469	0.8944
KNN	0.8333	0.8438	0.7500	0.9375	0.8333	0.8734	0.6875

2.5. Ligand-based meta-model

Table S4. Performance metrics for the trained logistic regression stacking model.

**Dataset**	**Accuracy**	**bACC**	**Sensitivity**	**Specificity**	**F1 Score**	**ROC AUC**	**MCC**
**Training**	0.8322	0.8308	0.8462	0.8154	0.8462	0.9223	0.6615
**Cross-validation mean**	0.8190	0.8201	0.8496	0.7906	0.8359	0.9184	0.6402
**Cross-validation SD**	0.0652	0.0694	0.0643	0.1219	0.0604	0.0621	0.1352
**Test**	0.9167	0.9125	0.9500	0.8750	0.9268	0.9594	0.8315

2.6. Docking-based classification

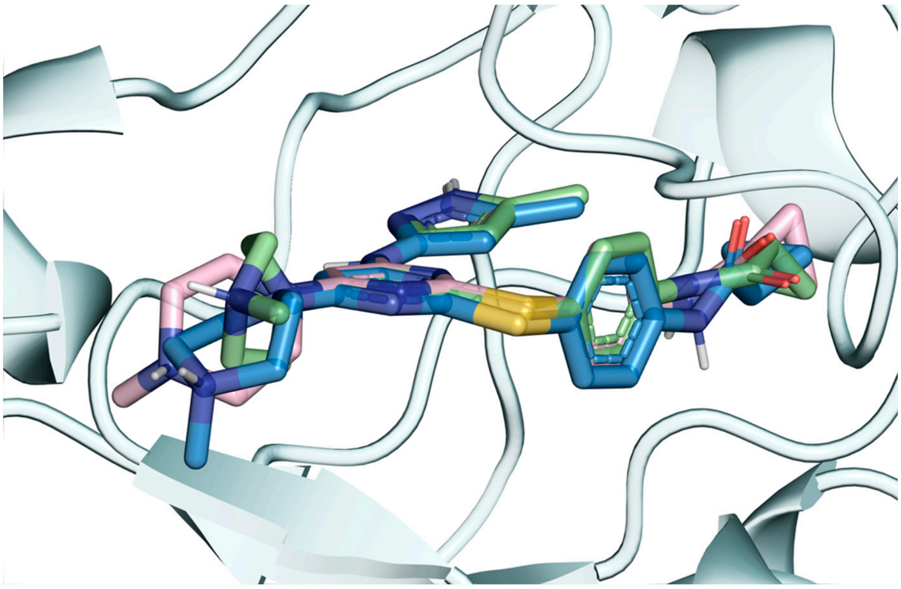

Figure S5. Superposition of binding poses predicted with Vina (green, RMSD = 2.3386 Å) and AD4 (pink, RMSD = 1.8497 Å) on co-crystal conformation of Vx-(blue).

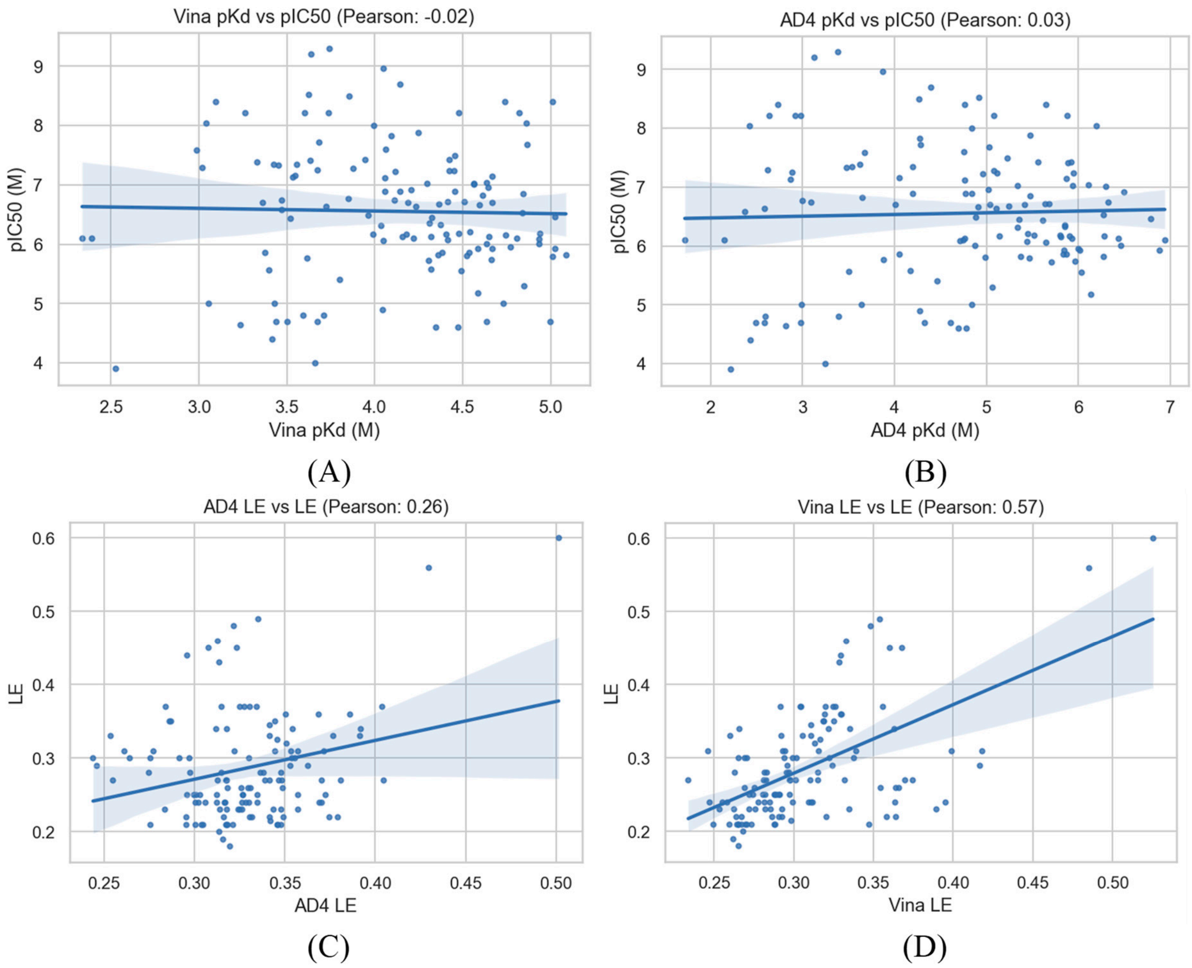

Figure S6. Correlation between docking results and experimental potency and efficiency values. (A) Experimental pIC50 values vs. predicted pKd values with AutoDock Vina (r = -0.0200, p = 0.7929); (B) experimental pIC50 values vs. predicted pKd values with AutoDock 4 (r = 0.0300, p = 0.7173); (C) experimental ligand efficiency (LE) values vs. predicted LE with AutoDock Vina (r = 0.2600, p = 0.0033); (D) experimental LE values vs. predicted LE with AutoDock 4 (r = 0.5700, p < 0.0001).

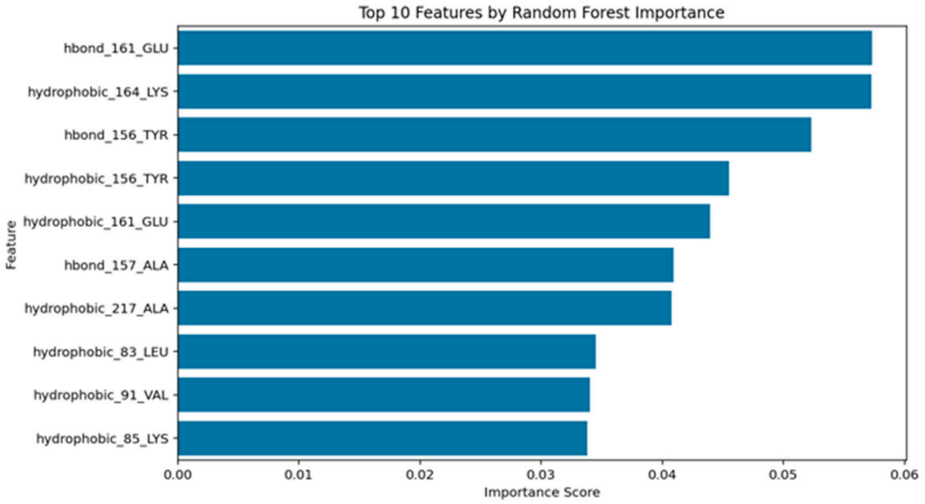

(A)

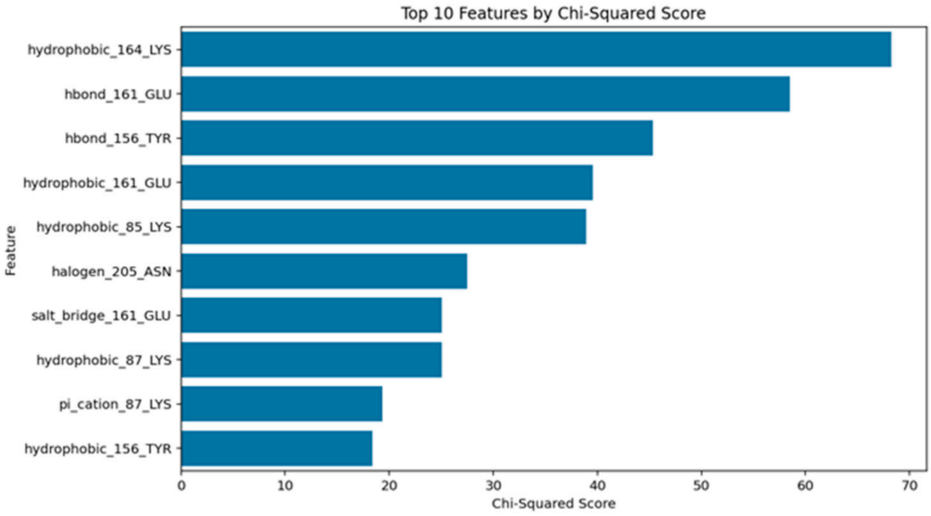

(B)

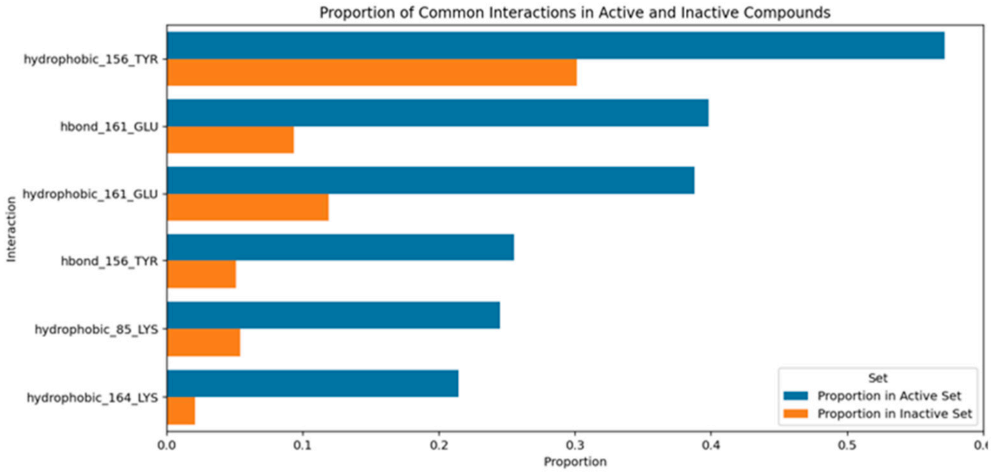

(C)

Figure S7. Selected interactions for model building. (A) RF-based selection of top 10 features; (B) top 10 features selected by Chi-squared test score; (C) proportions in active and inactive ligands for common interactions selected through both approaches.

Table S5. Performance metrics for the trained MLP model based on binding energies and interaction fingerprints.

Dataset	Accuracy	ROC AUC	Sensitivity	Specificity	bACC	F1 Score	MCC
Training	0.8508	0.9217	0.8462	0.8515	0.8488	0.6027	0.5546
Cross-validation mean	0.8234	0.8905	0.7821	0.8297	0.8059	0.5510	0.4964
Cross-validation SD	0.0748	0.0630	0.1775	0.0885	0.0896	0.1067	0.1331
Test	0.8973	0.9413	0.9500	0.8889	0.9194	0.7170	0.6896

2.7. Drug repurposing study

Table S6. Top 30 repurposable compounds ranked by predicted probabilities estimated using the docking-based MLP model.

Drug-Bank ID	Generic name	Group	P (MLP)	Binding energy (kcal/mol)	P (flex.)	Pred. pIC50	MCR_1_2	MCR_8	MCR_9	P (meta-model)
DB06660	Saredutant	I	0.9926	-11.40	0.5700	6.80	0	0	0	0.8572
DB00471	Montelukast	A	0.9895	-11.10	0.7800	6.35	0	0	0	0.7577
DB15190	GLPG-0259	I	0.9895	-11.10	0.3450	6.80	0	0	0	0.7246
DB11703	Acalabrutinib	A; I	0.9894	-11.09	0.3025	7.00	0	0	0	0.8066
DB15327	Abivertinib	I	0.9890	-10.46	0.3450	7.91	0	0	0	0.9885
DB11363	Alectinib	A; I	0.9872	-11.74	0.5550	6.69	0	0	0	0.8000
DB16851	Bentamapimod	I	0.9867	-11.33	0.3633	6.90	0	0	0	0.7936
DB03044	Doramapimod	I	0.9865	-11.31	0.3450	7.05	0	0	0	0.8499
DB18442	ABSK-091	I	0.9856	-9.82	0.1183	7.49	1	0	0	0.9127
DB15367	LY-2623091	I	0.9845	-12.28	0.3450	7.65	0	0	0	0.9741
DB16255	Nidufexor	I	0.9845	-10.64	0.4100	6.58	0	0	0	0.6284
DB12247	AZD-4547	I	0.9844	-9.78	0.1183	7.49	1	0	0	0.9127
DB05678	SLx-4090	I	0.9747	-11.29	0.0900	7.40	0	0	0	0.8708
DB17146	Abequolixron	I	0.9731	-10.17	0.3250	7.05	0	0	0	0.8438
DB12302	CP-724714	I	0.9729	-10.08	0.0900	7.48	0	0	0	0.8975
DB16253	Nemiralisib	I	0.9728	-11.60	0.3450	6.75	0	0	0	0.6933
DB11645	PF-4191834	I	0.9718	-10.87	0.7200	6.52	0	0	0	0.8103
DB00549	Zafirlukast	A; I	0.9716	-10.86	0.0900	7.30	0	0	0	0.8311
DB18715	Tolebrutinib	I	0.9707	-11.43	0.3025	6.94	0	0	0	0.7756
DB05076	Fenretinide	I	0.9681	-9.93	0.3450	6.92	0	0	0	0.7947
DB16843	ODM-203	I	0.9677	-10.93	0.3450	6.76	0	0	0	0.6969
DB16043	PF-06882961	I	0.9665	-12.46	0.3633	6.67	0	0	0	0.6510
DB18095	Edralbrutinib	I	0.9659	-12.48	0.3025	7.78	0	0	0	0.9797
DB13093	TAK-593	I	0.9654	-9.87	0.3450	7.25	0	0	0	0.9146
DB05424	Canertinib	I	0.9649	-10.39	0.4100	8.10	0	0	1	0.9963
DB12540	Lecozotan	I	0.9643	-11.51	0.2333	7.60	0	0	0	0.9558
DB06519	Edaglitazone	I	0.9631	-11.18	0.3025	6.90	0	0	0	0.7531
DB08387	Mardepodect	I	0.9617	-10.07	0.3450	6.94	0	0	0	0.8008
DB18307	VLX-1005	I	0.9596	-9.78	0.0900	7.36	0	0	0	0.8584
DB17191	Altiratinib	I	0.9521	-11.02	0.2425	7.89	0	0	0	0.9823

P – predicted probability; MLP – multi-layer perceptron; flex. – flexophores; I – investigational; A – approved.
